# Precision in a rush: Trade-offs between reproducibility and steepness of the hunchback expression pattern

**DOI:** 10.1371/journal.pcbi.1006513

**Published:** 2018-10-11

**Authors:** Huy Tran, Jonathan Desponds, Carmina Angelica Perez Romero, Mathieu Coppey, Cecile Fradin, Nathalie Dostatni, Aleksandra M. Walczak

**Affiliations:** 1 Ecole Normale Supérieure, PSL Research University, CNRS, Sorbonne Université, Laboratoire de Physique Théorique, Paris, France; 2 Institut Curie, PSL Research University, CNRS, Sorbonne Université, Nuclear Dynamics, Paris, France; 3 Department of Physics, University of California, San Diego, La Jolla, California, United States of America; 4 Department of Physics and Astronomy, McMaster University, Hamilton, Canada; 5 Institut Curie, PSL Research University, CNRS, Sorbonne Université, Physico Chimie, Paris, France; MRC-National Institute for Medical Research, UNITED KINGDOM

## Abstract

Fly development amazes us by the precision and reproducibility of gene expression, especially since the initial expression patterns are established during very short nuclear cycles. Recent live imaging of *hunchback* promoter dynamics shows a stable steep binary expression pattern established within the three minute interphase of nuclear cycle 11. Considering expression models of different complexity, we explore the trade-off between the ability of a regulatory system to produce a steep boundary and minimize expression variability between different nuclei. We show how a limited readout time imposed by short developmental cycles affects the gene’s ability to read positional information along the embryo’s anterior posterior axis and express reliably. Comparing our theoretical results to real-time monitoring of the *hunchback* transcription dynamics in live flies, we discuss possible regulatory strategies, suggesting an important role for additional binding sites, gradients or non-equilibrium binding and modified transcription factor search strategies.

## Author summary

Despite very limited time, organisms develop in reproducible ways. In the early stages of fly development the information about maternal signals is read out in a few minutes to produce steep and precise gene expression patterns. Motivated by recent live imaging experiments in fly embryos, we explore the consequences of the trade-off between a rushed but reproducible readout and a steep expression pattern on the regulatory modules of gene expression. We show that the current view of one anterior gradient morphogen binding to six binding sites is quantitatively inconsistent with the experimental data given the short readout time, suggesting other regulatory features.

## Introduction

**Fig 1:**
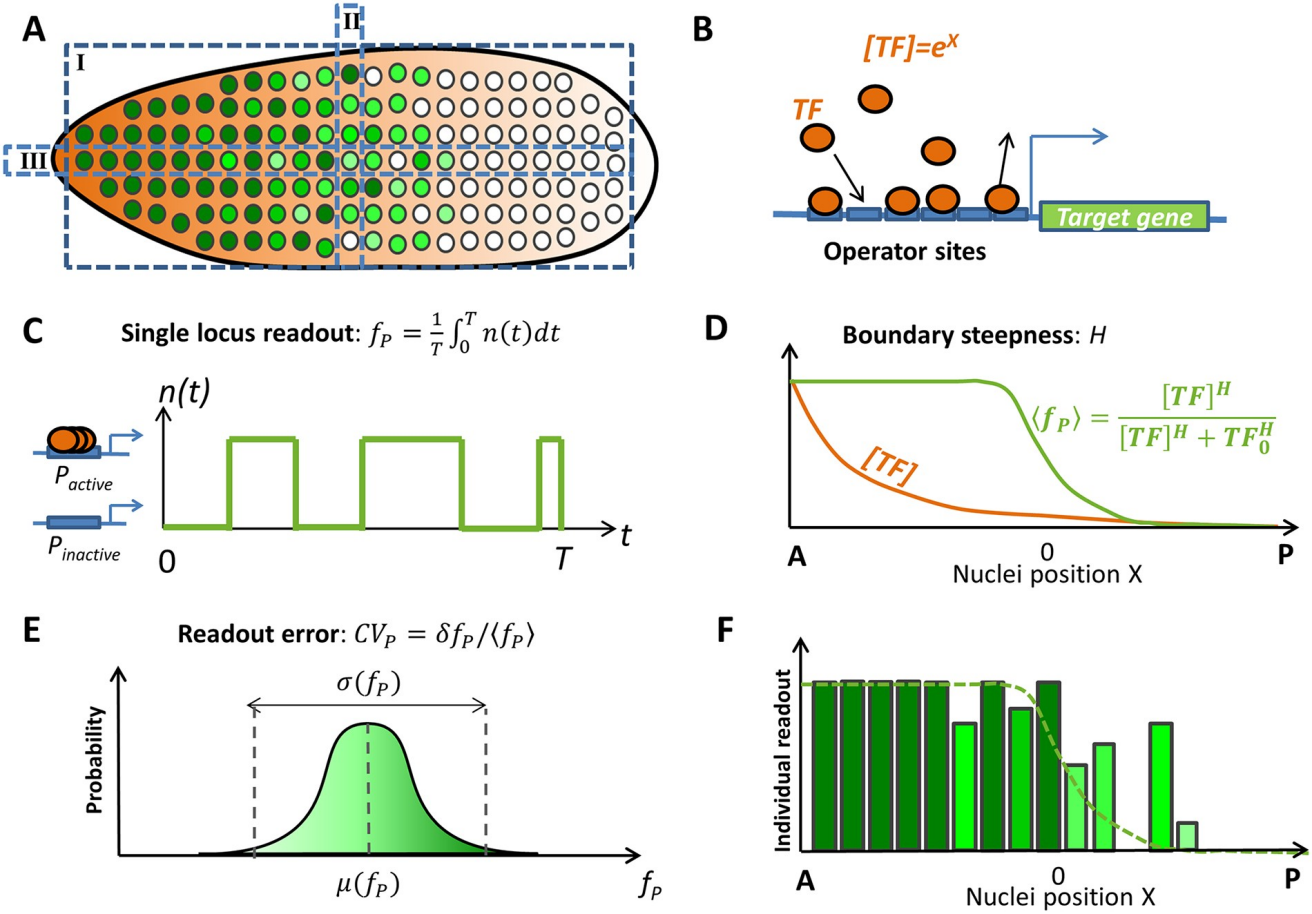
Setup of the problem: Features of the *hb* transcription pattern in early fly development. (A) A cartoon of the side section of the fly embryo in nuclear cycles (nc) 11-13. Nuclei at different positions along the anterior posterior (AP) axis express different concentrations of *hb* mRNA, represented by different shades of green (dark green denotes larger concentrations). The *hb* gene expression pattern can be studied from different perspectives: (I) Side view of the whole embryo. (II) Single columns of nuclei at similar position along the AP axis. (III) Single rows of nuclei along the AP axis. (B) The expression of *hb* mRNA from the *hb* gene is regulated by Bcd transcription factor (TF) binding. We consider a model of gene expression regulation via the binding and unbinding of Bcd proteins (orange) to multiple operator sites (blue) of the promoter. Bcd forms an exponentially decaying gradient along the AP axes and the concentration of Bcd TF in the nuclei depends on the position of the nucleus along the AP axis, *X*. (C) During each nuclear cycle each *hb* loci switches from periods of activation to inactivation by binding and unbinding Bcd TF. The distribution of these periods depends on the binding and unbinding rates of Bcd TF. Within the model, each *hb* loci produces a readout *f*_*P*_ defined as the average promoter activity level *n*(*t*) during the steady state expression interval *T* of the interphase of a given nuclear cycle. (D) By observing the whole embryo (perspective I in Fig. 1 A), we are able to calculate the average of the expression pattern *f*_*P*_ of the nuclei, 〈*f*_*P*_〉, as a function of the nuclei’s position along the AP axes (green line), from which the boundary steepness (denoted by *H*) is quantified by fitting a Hill function of Bcd concentration [*TF*] (orange line). *TF*_0_ denotes the concentration of Bcd TF at half-maximal expression. (E) By observing nuclei at similar position *X* along the AP axis (perspective II in Fig. 1 A), we can make a distribution of the readout, *f*_*P*_ and use it to calculate the readout errors *CV*_*P*_ in the single locus readout *f*_*P*_. *CV*_*P*_ is defined as the standard variation of the readout *f*_*P*_ divided by its mean. (F) The detailed expression pattern *f*_*P*_ obtained by observing a single row of nuclei (perspective III in Fig. 1 A) along the AP axis, depends both on the averaged pattern and the errors in the readout of this mean value.

During development reproducible cell identity is determined by expressing specific genes at the correct time and correct location in space in all individuals. How is this reproducible expression pattern encoded in the noisy expression of genes [1, 2], and read out in a short amount of time? We study this question in one of the simplest and the best understood developmental examples—the Bicoid-*hunchback* system in *Drosophila melanogaster*. In the fly embryo, the exponentially decaying Bicoid (Bcd) gradient [3–5] acts as a maternal source of positional information along the embryo’s Anterior-Posterior (AP) axis [6]. The *hunchback* (*hb*) gene extracts this positional information from the local Bicoid concentration and forms a steep binary-like expression pattern, observed as early as in nuclear cycle (nc) 10 (see Fig 1A) [3, 7–9]. This Hb pattern later becomes a source of positional information for the formation of other gap gene patterns [10, 11], forming the first step in the differentiation of cecullar phenotypes.

From real-time monitoring of the *hb* transcription dynamics [12, 13] using the MS2-MCP RNA-tagging system [14], we observed that from nc11 to nc13 the positional readout process of the *hb* gene is interrupted by mitosis, leaving a window of 5-10 minutes for gene expression in each cycle. Once the pattern stabilizes 2-3 minutes after mitosis, as we describe in detail in a companion experimental paper [13], the boundary between regions of high and low *hb* transcription is already steeper than even the Hb protein concentration profile in nc14 [13, 15, 16].

Several studies have proposed that the steep boundary between regions of high and low *hb* expression, given the smooth Bcd transcription factor (TF) gradient, is due to the cooperativity between the TF binding sites (Fig 1B) [7, 15, 17–20]. This cooperativity diversifies gene expression levels given small changes in the input [21–23]. Conventionally, the pattern steepness is represented by the Hill coefficient *H*. We define the *hb* gene readout as the *hb* gene transcription state of one locus in a single nucleus averaged over a given transcription window, *f*_*P*_, (Fig 1C). We can evaluate this quantity as a function of the TF concentration [*TF*] (Fig 1D), and thanks to the exponential nature of the Bcd gradient [15], uniquely associate a position along the AP axis to a Bcd concentration [TF]. The Hill coefficient is then estimated by fitting the mean readout value averaged over all nuclei at a specific position along the AP axis, 〈*f*_*P*_〉, to a sigmoidal function:

$$\left\langle f_{P}\right\rangle ∼\frac{{\left[TF\right]}^{H}}{TF_{0}^{H}+{\left[TF\right]}^{H}},$$ (1)

where *TF*_0_ is the Bcd concentration that results in half-maximal *hb* expression, $\langle f_{P}{|}_{\left[TF\right]=TF_{0}}\rangle =0.5$ (Fig 1D). *TF*_0_ defines the middle of the boundary, which we will call the mid-boundary point, that separates the highly expressing “ON” nuclei in the anterior region and the minimally expressing “OFF” nuclei in the posterior region of the embryo. Within a simple model where *hb* expression depends only on the binding and unbinding of Bcd to the *hb* promoter, the maximal steepness of the *hb* expression pattern was shown to depend on the number of operator binding sites in the promoter region of the gene *N*. Depending on whether this process conserves detailed balance or not, the maximal Hill coefficient is 2*N* − 1 or *N*, respectively [18].

These studies did not address whether such a steep boundary is achievable within the limited time window of 3 to 15 minutes in nuclear cycles 11-13, in which the Bcd concentration is read. The effects of the time constrained readout are further aggravated by the fact that transcription is stopped before and during each mitosis [13, 24], suggesting that the *hb* expression pattern needs to be re-established in each nuclear cycle. In addition, the intrinsic noise in chemical processes leads to inherent errors in the Bcd concentration readout [16, 25]. This noise results in a lower bound for the Bcd concentration readout error, defined as the standard deviation of the concentration of the measured molecule divided by its mean (Fig 1E), that depends on the readout integration time and the diffusion constant of ligand molecules [5, 26–29]. Extending the original work that considered a single or an array of non-interacting receptors [5, 26], other work pointed out that cooperativity from receptor arrays increases the readout noise [30, 31]. Given these effects, it is unclear how the readout precision of the Bcd concentration (or nuclei position) changes quantitatively given the highly cooperative readout process by the promoter observed as the steep *hb* expression pattern [9, 15, 16] (Fig 1F) and what are the consequences for the ability of neighboring nuclei to take on different cell fates. In this work, we investigate how the constraints coming from short cell cycles affect the steepness and errors in the *hb* expression pattern.

## Results

### The model

In the early stage of development, the *hb* transcription pattern is steep, despite relying mostly on the exponential Bcd gradient as the source of positional information [8]. It was hypothesized that Bcd molecules can bind cooperatively to the many Bcd binding sites on the *hb* promoter, enabling the gene to have diverse expression levels in response to gradual changes in the Bcd concentration [7, 18]. We use a simple model of gene expression regulation by binding of Bcd transcription factors (TF) to the operator sites (OS) of the target promoter [18] (Fig 1B, Fig A in S1 Text). The promoter activity depends on the occupancy state of the operator sites and we consider different activation schemes, which we specify below. The binding rates are functions of the position-dependent TF concentration and we further assume their value is bounded by the promoter search time of individual TFs (see S1A Text). The promoter readout, *f*_*P*_, is defined as the mean of the promoter activity level *n*(*t*), calculated from the temporal average of the promoter state *n*(*t*) over the steady state expression interval *T* of a given nuclear cycle interphase (see Fig 1C).

We first focus on a simplified version of the general model of gene regulation for binding of Bcd TF to the *N* OS [18], where all the binding sites of the target promoter are identical. This assumption gives a Markov model of TF binding/unbinding to the many identical OS of the target promoter:

$$P_{0}\overset{k_{1}\left[TF\right]}{\underset{k_{-1}}{⇌}}P_{1}\overset{k_{2}\left[TF\right]}{\underset{k_{-2}}{⇌}}P_{2}\ldots \overset{k_{N}\left[TF\right]}{\underset{k_{-N}}{⇌}}P_{N},$$ (2)

where *P*_*i*_ denotes the promoter state with *i* bound OS and *N* − *i* free OS. [*TF*] is the relative Bcd TF concentration with respect to that at the mid-boundary position. Since Bcd concentration decays exponentially along the embryo AP axis, we estimate the relative nuclei position *X* measured in terms of the gradient decay length from the TF concentration (*X* = ln([*TF*]), such that at mid-boundary *X* = 0 and [*TF*] = 1. The binding and unbinding of TF to the promoter occur with rate constants *k*_*i*_ and *k*_−*i*_. If all the rates are non-zero, all reactions are reversible and Eq 2 defines an equilibrium model.

Throughout the paper, we randomize the binding and unbinding rates to explore the behavior of the model (see Methods for details). When comparing models with different parameters we rescale unbinding rate values *k*_−*i*_ in order to keep the binding rate at the mid-boundary position constant. In order to best align to experimental observations, we estimate this fixed binding rate at −5% embryo length (EL) (−50% EL and 50% EL are the embryo’s anterior and posterior poles), which is the typical boundary position in the analyzed wild type embryos [13] (see S1B Text).

We first consider the “all-or-nothing” case, i.e. the promoter is active when the OS are fully bound by TF (*P*_*N*_ ≡ *P*_active_), although the qualitative conclusions remain the same for the “*K*-or-more” scenario [18, 30], where the promoter is active if at least *K* sites are occupied (see the next section). At steady state, we find the probability that the promoter is in the active state given the nucleus position *X* (see S1A Text):

$$P\left(P_{\text{active}},X\right)=\frac{{\tilde{K}}_{N}e^{N\cdot X}}{\sum_{i=0}^{N}{\tilde{K}}_{i}e^{iX}},$$ (3)

where for convenience of notation we define the effective equilibrium constant ${\tilde{K}}_{i}=\prod_{j=1}^{i}k_{j}/\prod_{j=i}^{i}k_{-j}$ and ${\tilde{K}}_{0}=1$. We assume the target gene transcription rate at steady-state is proportional to the probability of the promoter to be in the active state (Eq 3).

The steepness of the expression pattern is quantified by the Hill coefficient *H* [32], calculated as the slope of the expression pattern at the mid-boundary position (see S1C Text) $H=N-\left(\sum_{i=1}^{N-1}i\cdot {\tilde{K}}_{i}\right)/{\tilde{K}}_{N}$. *H* is bounded from below by 1, and from above by *N*—the OS number, confirming previous results [18]. Maximum steepness (*H* = *N*) is achieved when the system spends most of the time in the fully free (*P*_0_) or fully bound states (*P*_*N*_) while *H* = 1 when the system spends most of the time in highly occupied states *P*_*N*−1_ and *P*_*N*_ (see S1D Text).

Lastly, we also consider a full non-equilibrium binding model (defined in Fig A in S1 and S1G Text), in which not all binding reactions are reversible, and reversible equilibrium models with two different types of TF factors (defined in S1H Text). To explore the properties of all of these models, we solve the time dependent equations of motion for the stochastic binding models numerically and, when possible analytically in steady state, considering different expression schemes (“all-or-nothing” and “*K*-or-more”), different numbers of TF binding sites and randomizing binding and unbinding parameters (see Methods).

### The expression pattern formation time

**Fig 2:**
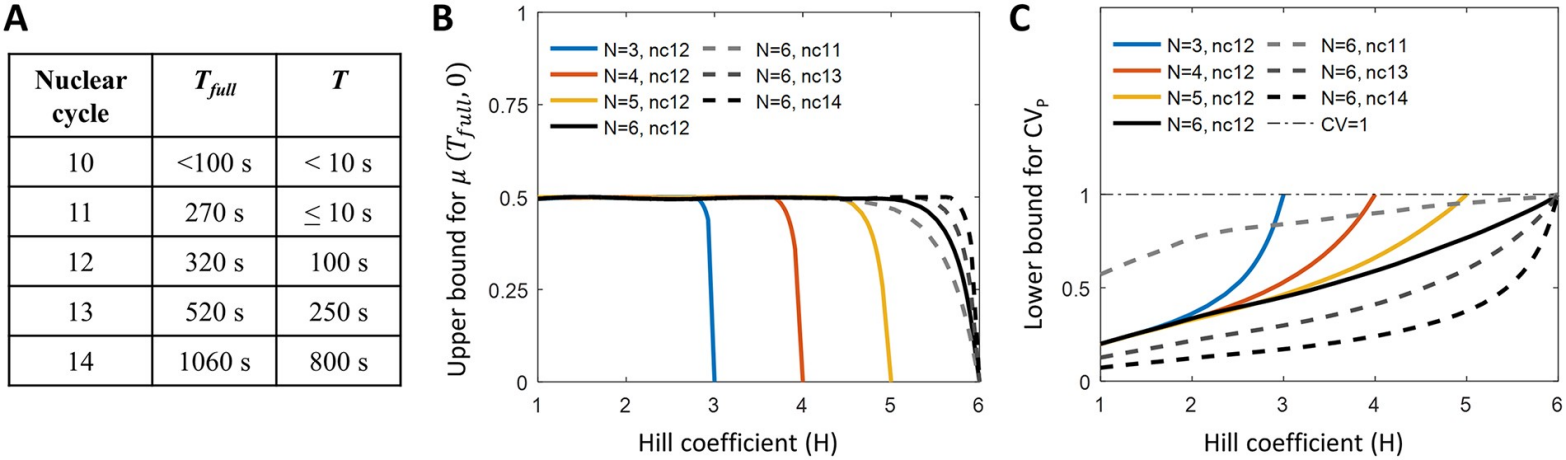
Equilibrium model predictions for the pattern formation time and readout error. (A) The early nuclear cycles have a short interphase *T*_full_ and even shorter steady state periods *T* when the average transcription rate is neither increasing after, nor decreasing before, transcription shut-off during mitosis. The total transcription window *T*_*full*_ and the time window when the transcription rate is at steady state *T* of *hb* transcription in nuclear cycle 10, 11, 12, 13 and early nuclear cycle 14 (before cellularization) at 25°C obtained from 8 MS2-MCP movies [13]. The short periods of transcription inactivity right before and after mitosis are excluded. (B) Steep steady state expression profiles (large *H*) cannot be reached in short nuclear cycles. Since transcription is shut-off during mitosis, the sigmoidal expression pattern (as in Fig 1D), characterized by the mean promoter activity in nuclei positioned at mid-boundary *μ*_*P*_(*T*_full_, 0) = 0.5, needs to be re-established in each nuclear cycle. We randomize the binding and unbinding rates of the equilibrium model to calculate the upper bound for the mean promoter activity in nuclei positioned at mid-boundary *μ*_*P*_(*T*_full_, 0), and the corresponding Hill coefficient *H*, for varying OS number *N* and nuclear cycle duration *T*_full_. *μ*_*P*_(*T*_full_, 0) < 0.5 indicates the steady state expression profile could not be reached within the nuclear cycle duration. (C) Steep expression profiles (large *H*) correspond to equilibrium binding models with larger readout errors of the mean activity of the nuclei at the mid-boundary position. The readout error decreases with nc duration. Randomizing parameters of the equilibrium model we plot the lower bound for the readout error of the mean activity of the nuclei, *CV*_*P*_, defined as the standard variation of the readout *f*_*P*_ divided by its mean, for varying OS number *N* and steady state-period *T*. The bounds in (A-B) are calculated numerically from ∼50000 data points of the solutions of dynamical equations of the equilibrium model (S1D Text) with *N* OS, each corresponding to a randomized kinetic parameter set.

The *hb* expression pattern in the early phase of development is always formed under rigorous time constraints: the total time of transcription during an interphase of duration *T*_full_ varies in nc 10-13 from ∼100 seconds to ∼520 seconds (Fig 2A). During mitosis, Bcd molecules leave the nuclei and only re-enter at the beginning of the interphase [5]. The steep expression pattern takes time to reestablish. Assuming that at the beginning of the interphase all OS of the *hb* promoter in all nuclei are free, the mean probability *μ*_*P*_(*t*, *X*) for the promoter to be active at position *X* at time *t* following the entering of the TF to the nuclei is initially large only in the anterior of the embryo (see Fig C in S1 Text). By propagating the time dependent equations of motion for the stochastic equilibrium binding models (see S1E Text) in time, we see that with time *μ*_*P*_(*t*, *X*) increases also in other regions of the embryo, to reach its steady-state form (*P*(*P*_active_, X)) with a border between low and high expressing nuclei that defines the mid-boundary position.

Whether the interphase duration *T*_full_ in a given nuclear cycle is long enough for the system to reach steady state depends on the parameters of binding and unbinding of the TF to the operator sites. However, the binding and unbinding rates also determine the expression pattern steepness, leading to constraints between expression pattern steepness and formation time. Considering the “all-or-nothing” equilibrium model, when the promoter is active (*P*_*N*_ ≡ *P*_active_), any unbinding from the promoter inactivates the promoter. We find that a steep expression pattern requires the promoter to stay in the active state for a long time or to be regulated by binding of TF to many OS (see S1D Text):

$$H\leq N-\left(N-1\right)\frac{\tau_{\text{bind}}}{\tau_{\text{active}}},$$ (4)

where *τ*_active_ = (*k*_−*N*_)^−1^ is the mean time for the promoter to switch from the active state to the inactive state and *τ*_bind_ = (*k*_*N*_)^−1^ is the mean time for a TF to bind to the last unoccupied OS. The maximum value of *H* is reached when the promoter spends most of the time in the *P*_0_, *P*_*N*−1_ and *P*_*N*_ states (see S1D Text). This limit corresponds to very slow promoter switching, *τ*_active_ ≥ *τ*_bind_, similarly to conclusions obtained for cell surface receptors [30]. With typically considered parameters for the Bcd-hb system [9, 15, 33], *τ*_bind_ ∼ 4*s* and the *H* ∼ *N* limit in Eq 4 corresponds to *τ*_active_ ≫ 4 s (see S1D Text). Even if the currently available estimates for the value *τ*_bind_ prove inaccurate, the qualitative conclusion about slow promoter switching will remain unchanged.

Given the limited interphase duration in nc 11, *τ*_full_ ∼ 270s (Fig 2A), randomizing parameters of the equilibrium model (Eq 2) shows that a steep steady-state expression pattern cannot be established during the interphase: the upper bound for the mean promoter activity level *μ*_*P*_(*T*_full_, 0) at the mid-boundary position (*X* = 0) at the end of the interphase of duration *T*_full_ is less than the steady state value of 0.5 for kinetic parameters giving large Hill coeffcients *H* (Fig 2B). For long interphases (*T*_full_ ≥ 100 s), all patterns but those close to the maximum allowed steepness of *H* ≈ *N* reach steady state. For *H* ≈ *N*, Eq 4 imposes large *τ*_active_, which means there are not enough binding and unbinding events to achieve the steady state expression pattern with *μ*_*P*_(*T*_full_, 0) ∼ 0.5 at the boundary.

Generalizing the model to allow for non-equilibrium binding (Fig A in S1 Text) increases the possible Hill coefficients above *H* > *N* = 6, but does not alleviate their inaccessibility within the considered nuclear cycles 11-13 (Fig L in S1 Text). Given the observed steep boundary *H* ∼ 7 in nuclear cycles 11-13 [13, 16] and the relatively short interphase duration (*T*_full_ ∼ 520s in nc 13, see Fig 2A), it seems unlikely that the steep steady state boundary is reached in early fly development with only the *N* = 6 known Bicoid operator sites of the proximal *hb* promoter [7, 19]. Nevertheless the steady state results give a best case scenario for readout error estimates so we focus on an equilibrium steady state system in the next section. We then extend the arguments to out-of-equilibrium binding.

### The single locus readout error at steady-state

Even when the mean promoter dynamics over the nuclear population has reached steady-state, each individual gap gene in each nucleus must independently read the positional information and express mRNA in a way to ensure the transcription pattern’s reproducibility. The promoter in each nucleus switches between an active and an inactive state *n*(*t*) = 0, 1 (Fig 1C). The reproducibility of the transcriptional readout $f_{P}=\frac{1}{T}\int_{t=0}^{T}n\left(t\right)dt$ at the mid-boundary position in steady state is described by the nuclei-to-nuclei readout error of the mean activity of the nuclei *CV*_*P*_ = *δf*_*P*_/〈*f*_*P*_〉, where the average 〈〉 is over nuclei at the same position *X* = 0 calculated during the steady state expression window *T* in a given nuclear cycle (Fig 1C and 1E, see S1E Text).

Randomizing binding parameters in the equilibrium model (Eq 2) we see that the lower bound for the nuclei-to-nuclei readout error, *CV*_*P*_, increases with increasing Hill coefficient *H* and decreases with the nc duration (Fig 2C). A steep pattern requires slower promoter switching dynamics (Eq 4), which results in less independent measurements that take part in the single locus readout during each interphase. Therefore, the steeper the pattern, the larger the nuclei-to-nuclei readout error in the expression pattern due to the increased variability in the readouts, *f*_*P*_, between different nuclei [34]. When the steepness *H* approaches its upper bound limited by the maximum number of binding sites *N*, due to a small number of switching events during the interphase, the distribution of readout *f*_*P*_ approaches a Bernoulli distribution with *p* = 0.5 with the relative error always equal to $\sqrt{(1-p)/p}=1$, regardless of *T* and *N*. The decrease in readout error at small steepness depends on the length of the nuclear cycle (Fig 2C). For very short cycles (i.e. *T* < 10 s), only non steep patterns (*H* ≤ 2) are able to significantly reduce the readout errors. For long interphases (*T* > 100 s), significant reduction in readout errors can be achieved with steep patterns (*H* ∼ 5), and further decreasing *H* yields little improvement in reducing the readout error (Fig D in S1 Text).

In our models, *τ*_bind_ is the only external time scale in the problem. We assume it is set by diffusion (S1A Text) and all other timescales (e.g. the time to establish the steady state expression pattern, the value of *τ*_active_ that will minimize the time to establish the steady state profile) depend on it. If our estimate of *τ*_bind_ ∼ 4*s* is inaccurate and differs by orders of magnitude, then the conclusions about not being able to establish the steep steady state expression pattern may not hold. However the point of the analysis presented in this section remains valid—steep expression profiles result in large nuclei-to-nuclei variability.

### Positional resolution

The above analysis uncovers a trade-off between the readout error and steepness of the expression pattern at the boundary: the steeper the boundary, the larger the minimal nuclei-to-nuclei variability, quantified as the readout error (Fig 2C). Additionally, while long nuclear cycles seem desirable both to obtain the observed steep expression patterns and decrease nuclei-to-nuclei variability, the nuclear cycles 11-13 during which these steep patterns are experimentally observed [13] are very short (Fig 2A). In light of the experimental facts, steep expression patterns seem like an obstacle to reducing readout errors.

The trade-off between the expression pattern steepness and the nuclei-to-nuclei variability suggests that neither of these features alone can be used as the sole criterion for a reproducible pattern. This observation is not surprising given that these features emerged from looking at the embryo from two different perspectives (Fig 1A): the expression pattern steepness is perceived from an external observer’s perspective when looking at the *whole* embryo at a fixed time (Fig 1D), while the readout error is calculated by comparing nuclei at a similar position along the AP axis averaged over time (Fig 1E). These features are likely to be unobtainable to individual nuclei (Fig 1F), in which the decisions about transcription are made, since they require averaging or comparing the readout of different nuclei.

**Fig 3:**
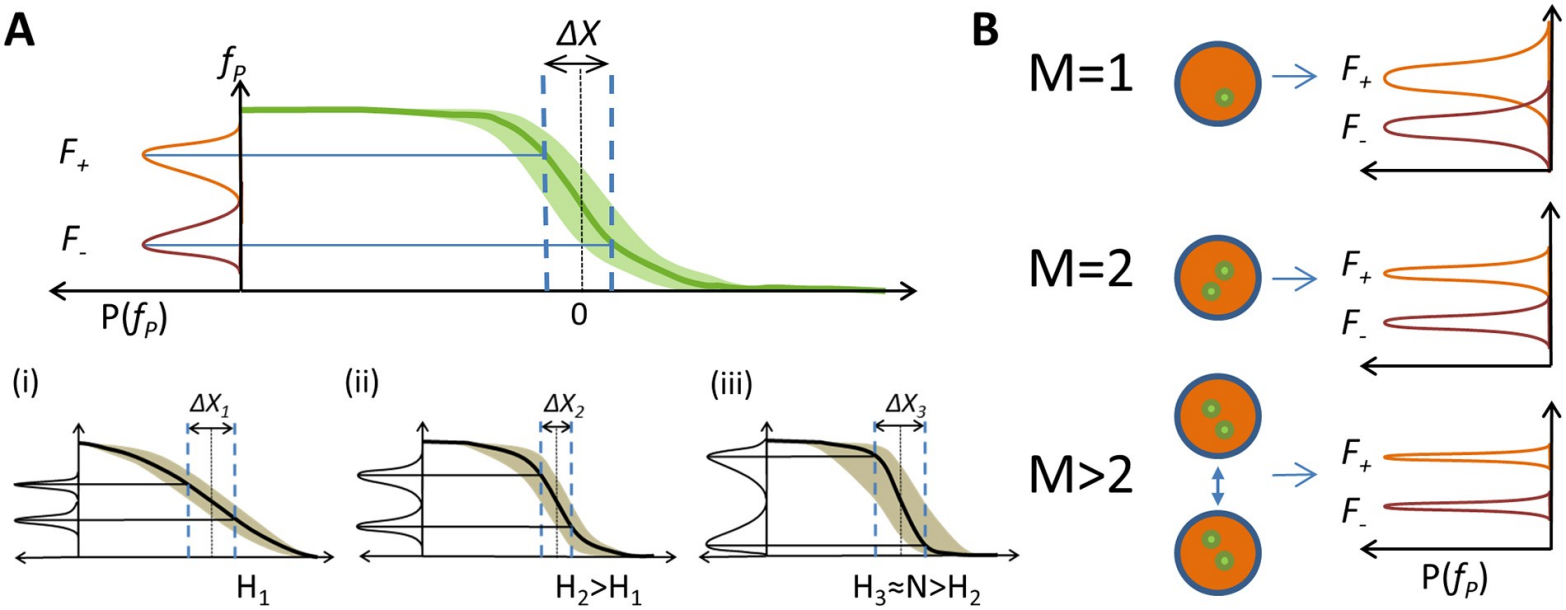
The positional resolution of the pattern. (A) We use the positional resolution Δ*X* to describe how well nearby nuclei can readout discernible inputs. *F*_+_ and *F*_−_ are the positional readouts in individual nuclei positioned at ±Δ*X*/2 from the mid-boundary point (*X* = 0). Positional resolution, Δ*X*, is the minimal distance between nuclei that make distinct readouts at steady state *P*(*F*_+_ ≤ *F*_−_) ≤ 0.05. Positional resolution results from a trade-off between the pattern steepness and the readout error: (i-iii) a cartoon representation of the trade-off for a flat pattern (low H, i), pattern of intermediate steepness (ii) and a steep pattern (high H, iii). Both (i) and (iii) have a large value of positional resolution. At low H (i), the readout errors are low but the mean readout values are very similar. At high H (iii), the mean readout values are different but the readout errors are large. The best positional resolution is reached with an intermediate H (ii). (B) Each nucleus readout is the average of M independent single locus readouts: *M* can be 1 (there is one copy of the gene as is the case in a heterogeneous gene construct), 2 (there is one gene copy on each chromosome as is the case of the WT embryo) or greater (nuclei at the same position can communicate by diffusion of readout molecules [35, 36] and the readout is the result of spatial averaging). As *M* increases, the readout error of the nuclei decreases due to spatial averaging.

In order to better understand the readout of reproducible cell fates from the perspective of an individual nucleus in the fly embryo, we use the positional resolution of the expression pattern, Δ*X* [9, 35], defined as the minimum distance between two nuclei located symmetrically on the two sides of the mid-border position *X* = 0 that have distinct readout levels in steady state (Fig 3A). Specifically, if *F*_+_ and *F*_−_ are the distributions of mRNA concentrations in two nuclei at positions +Δ*X*/2 and −Δ*X*/2 (see S1F Text), we define the positional resolution Δ*X* such that the probability of a false positive readout is small, *P*(*F*_+_ ≤ *F*_−_) ≤ 0.05. Positional resolution is a distance measure that we report in length units of % egg length (EL) or nuclei widths, where one nucleus width corresponds to 2% EL. The width of one nucleus (2% EL) sets a natural resolution scale for the problem—the embryo cannot achieve a better resolution than that of one nucleus. While positional resolution tells us how well a nucleus can distinguish its position from that of other nuclei, it is not a measure of information between the position along the AP axis and Bicoid concentration, such as the previously proposed positional information [37, 38]. The term positional resolution is borrowed from optics, and the higher the resolution the better, since it corresponds to a smaller minimal distance between nuclei that make distinct readouts. To avoid confusion, in the text we refer to the minimal value of the positional resolution Δ*X* as the best case scenario when nuclei separated by a small distance make discernable readouts.

The trade-off between the pattern steepness and the readout error translates into constraints on the positional resolution. For a flat expression pattern (low *H*, Fig 3A, panel (i)), *F*_+_ and *F*_−_ have a small difference in their mean value, which makes it hard to differentiate the mRNA concentration in closely positioned nuclei, but the variance around their mean is also small. On the other hand, with a very steep pattern (Fig 3A, panel (iii)), *F*_+_ and *F*_−_ have a big difference in their mean mRNA expression but also an increased variance, due to the increased readout errors in particular nuclei. An intermediate Hill coefficient offers the best positional resolution (Fig 3A, panel (ii)).

To evaluate the positional resolution for a given pattern steepness *H* and steady state expression interval *T* in a given nuclear cycle we randomize all the binding/unbinding parameters for a promoter with *N* = 6 OS—a number inspired by the number of Bicoid binding sites found on the *hb* promoter [7, 19]. We identify the parameters that give the smallest *CV*_*P*_ to ensure the smallest Δ*X*. *CV*_*P*_ and Δ*X* are tightly correlated (Fig I in S1 Text) but *CV*_*P*_ is faster to evaluate.

**Fig 4:**
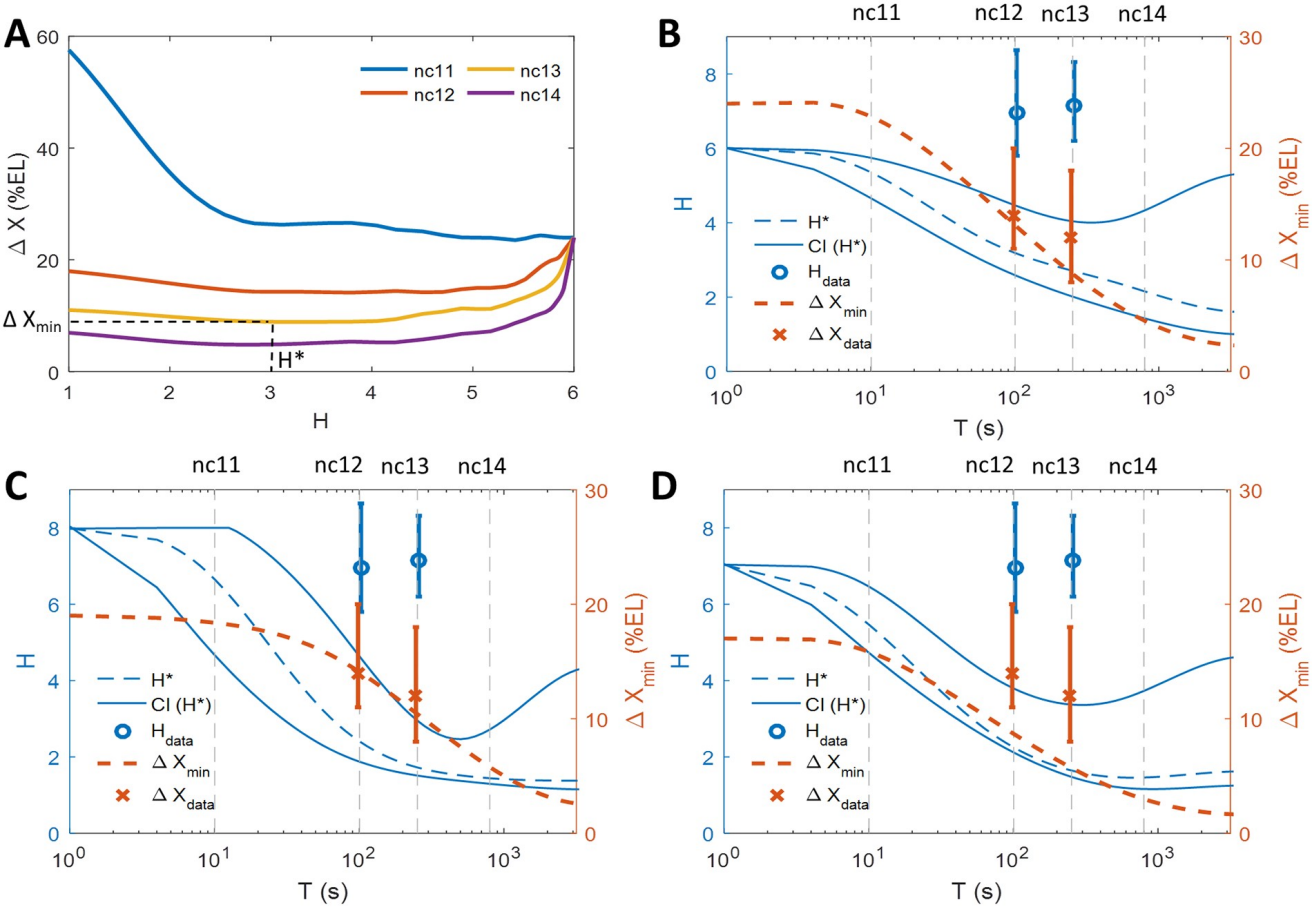
The positional resolution of the expression pattern for different nuclear cycles and regulatory models. (A) Positional resolution calculated from the equilibrium binding site model with randomized kinetic parameters that give different values of the expression profile steepness *H* for *M* = 1. The colored lines show the results for parameters that give the smallest readout error *CV*_*P*_ from a set of randomized parameters for the steady-state window *T* in nc 11-14 (Fig 2A). The curves are smoothed using cubic spline interpolation for better visualization. For each nuclear cycle we find the optimal Hill coefficient H* that results in a model with the smallest value of positional resolution, Δ*X*_min_. (B-D) The range of optimal Hill coefficients H* (dashed blue line) that yield the lowest value of the positional resolution (obtained as described in Fig. 4A) as a function of the steady-state readout duration *T* for the equilibrium *N* = 6 model (defined in Eq 2 and S1D Text) (B); the hybrid *N* = 6 non-equilibrium model with 3 equilibrium and 3 non-equilibrium OS (defined in S1G Text) (C); and the two mirror TF gradient model (defined in S1H Text)(D). Around the optimal Hill coefficients H* that give the minimal value of the positional resolution Δ*X* (dashed blue line) we also plot the range of Hill coefficients that come from models resulting in Δ*X* = Δ*X*_min_ ± 2% EL, allowing for a tolerance interval for the positional resolution (solid blue lines). The curves are smoothed by cubic spline interpolation for better visualization. Also shown is the lowest achievable value of the positional resolution in the numerical randomization experiment for varying *T* (dashed orange line). The results are obtained assuming a diffusion limited estimate for *τ*_bind_ = 4*s*. The theoretical results for *M* = 1 for all models are compared to the empirical Hill coefficient *H*_data_ (blue circles with error bars) and positional resolution Δ*X*_data_ (orange crosses with error bars) extracted from MS2-MCP live imaging data in nc 12 and nc 13 [13] (see S1I Text). The error bars correspond to 95% confidence intervals. In general, only the non-equilibrium model with *N* = 6 is able to produce both Hill coefficients and Δ*X* values observed in experiments. However, assuming *t*_bind_ = 4*s*, even the non-equilibrium model cannot achieve the experimental values of the Hill coefficient during the time *T* of nc 12-13.

For short nuclear cycles (small *T*), there are hardly any promoter switching events during the readout time window and the readout error *CV*_*P*_ ∼ 1 for all values of *H* (Fig 2C). In this case, the positional resolution is mainly governed by the increase in the difference between *F*_+_ and *F*_−_, with increasing Hill coefficients *H*, which leads to a decrease in Δ*X* (Fig 4A). As *T* lengthens, the value of the positional resolution Δ*X* for small Hill coefficients decreases with increasing *H*, due to the reduced readout error from averaging promoter switching events, until a certain value, Δ*X*_min_(*T*). As *H* approaches *N*, the readout error increases drastically since *CV*_*P*_ → 1 (Fig 2C). As a result, the value of the positional resolution Δ*X* increases and converges to a fixed value Δ*X*_*N*_ ≈ 24% EL independently of *T* (see S1F Text).

We asked what values of Hill coefficients give the best ability for close-by nuclei to distinguish their position along the AP axis, and whether these values change with the duration of the nuclear cycle. To this end for each steady state transcription period *T*, we read-off the minimal value of the positional resolution Δ*X* predicted by our model, Δ*X*_min_(*T*), from Fig 4A to produce the orange line in Fig 4B. We also plot the optimal Hill coefficients corresponding to the minimal value of the positional resolution, H* = *H*(Δ*X*_min_) as a function of *T*—the dashed blue line in Fig 4B. We found that the Hill coefficients H* that guarantee the best positional resolution decrease with the nuclear cycle duration. Since the embryo need not be performing an optimal positional readout, we found the range of Hill coefficients that allow for a margin of error of about one nucleus (2% of the embryo’s length). The choice of 2% of the embryo’s length is arbitrary, yet motivated by the observation that close-by nuclei do make different readout and this assumption allows us to explore the properties of the model. The solid blue lines in Fig 4B denote a confidence interval of *H* that results in a positional resolution within 2% of the embryo’s length of the optimal value.

We see that for short nuclear cycles (up to nc 11), the embryo can best discriminate readouts when producing a very steep pattern (intersect of dashed blue and dashed gray nc 11 line in Fig 4B). For longer nuclear cycles (12 and 13), a narrow range of moderately steep profiles (H* between 2 and 5) result in the smallest values of positional resolution (intersect of dashed blue and dashed gray nc 12 and nc 13 line in Fig 4B). As the steady state transcription period *T* increases, Δ*X* becomes very small for expression profiles with a wide range of *H* and the constraint on H* is relaxed (blue solid lines for large *T* in Fig 4B). In this case a discernible readout owing to small values of positional resolution can be reached even for very flat expression profiles, since time averaging alone can result in reproducible readouts.

To compare the model predictions to experimental data, in Fig 4B–4D we plot the Hill coefficient (blue dot) and positional resolution (orange cross) obtained from the analysis of MS2-MCP imaging of fly embryos in nc 12 and 13 [13]. To avoid variability in the Bcd concentration between embryos, the analysis was performed by aligning 8 embryos in nc 12 and 4 embryos in nc 13 at the point of their half-maximal value of the integral fluorescence intensity. The Hill coefficients are calculated by fitting a sigmoidal curve to the mean normalized fluorescence intensity averaged over nuclei at similar positions as a function of the AP axis from data combined from multiple embryos (see S1I Text for details). To calculate the positional resolution we take the normalized fluorescence intensity as the readout of each nucleus within a 5% EL bin around *X* = 0 and follow the procedure described above and in S1I Text. The errors bars in Fig 4B–4D for both observables represent the 95% confidence intervals. The experimental positional resolution is Δ*X*_data_ ∼ 14% EL (confidence interval from 11% to 20%) in nc 12 and Δ*X*_data_ ∼ 12% EL (confidence interval from 8% EL to 18% EL) in nc 13. The experimental Hill coefficient value is *H*_data_ = 6.9 (confidence interval [5.80, 8.64], *p* < 0.05) in nc 12 and *H*_data_ = 7.1 (confidence interval [6.20, 8.32], *p* < 0.05) in nc 13. The experimental positional resolution in these early nuclear cycles is well predicted by an equilibrium model with *N* = 6 binding sites (orange dashed line and orange dots in Fig 4B), but the experimental Hill coefficient is larger than the model prediction (blue dashed line and blue dots in Fig 4B).

### An effective treatment of spatial averaging

To explore the effect of multiple gene copies on positional resolution we generalize the model with *M* = 1 that describes the readout from a heterogenous gene to *M* = 2, which describes a homogenous gene readout made independently in one nucleus (Fig 3B). Although the density of nuclei does increase as nuclear cycles progress, assuming that each nuclei is making an independent measurement of the Bcd concentration (*M* = 1 for a heterogenous gene or *M* = 2 for a homogenous gene), the minimal distance between nuclei that make a distinct readout measured in units of length, will not change. However, spatial averaging of the readout concentration changes the positional resolution Δ*X*. In our model we account for spatial averaging of mRNA in the cytoplasmic space coming from different nuclei [35] in an effective way by assuming that the readout in a given nucleus is the average of more than two genes (*M* > 2, Fig 3B).

The results for the mRNA readout in a nucleus coming from a single expressing gene copy (*M* = 1—a heterozygous fluorescent marker such as in recent MS2-MCP experiments [12]) hold for a readout coming from more gene copies (*M* > 1, Fig 3B, Fig H in S1 Text). As expected, averaging over many gene copies further reduces the readout noise and slightly decreases the minimal value of positional resolution (Fig H in S1 Text). We opt for an effective treatment of spatial averaging at the mRNA level, since the scale of the phenomenon has not yet been quantified in experiments in nc 11-13 and a more detailed model would require making arbitrary assumptions. In general, the strength of the averaging effect is likely to increase with time, as the nuclei density increases and the nuclear cycles get longer. Our model does not capture these time dependent effects because the role of averaging is likely to be limited during the very short time of ∼2 minutes when the steep expression pattern is established [13].

### The non-equilibrium model

Comparing the results of the equilibrium binding site model to experimental observations, we note that the steepness values obtained in experiment (*H*_data_ ∼ 7) cannot be reached by an equilibrium model with the identified *N* = 6 Bcd binding sites on the proximal *hb* promoter. Estrada *et al*. [18] noted that this threshold of *H* = *N* can be overcome with a non-equilibrium binding model. We considered a full non-equilibrium model for *N* = 3 (Fig A in S1 Text) and a hybrid model for *N* = 6 due to the computational complexity of performing a parameter scan of a full *N* = 6 non-equilibrium model. In the hybrid model, the promoter has 3 OS whose interactions with TF are in equilibrium and 3 OS whose interactions with TF are out-of-equilibrium (see S1G Text).

The boundary steepness within these models can be larger than the number of operator sites (*H* ≤ 5 for the *N* = 3 case, Fig K in S1 Text, and *H* ≤ 8 for the hybrid *N* = 6 case, Fig L in S1 Text). However, the conclusions drawn from the equilibrium model are still valid even for *H* > *N*. Large Hill coefficients result in larger readout errors (Figs K and L in S1 Text). For the *N* = 6 hybrid model, the value of the positional resolution is minimal for large *H* only for very short interphase durations, and for longer interphase durations lower Hill coefficients give smaller Δ*X* (Fig M in S1 Text). For interphase durations found in the fly embryo, intermediate Hill coefficient values, 2 ≤ *H* ≤ 5, provide the best positional resolution of ∼ 6 to 10% EL or 6 to 7 nuclei lengths (Fig 4C), smaller than the observed experimental values of ∼14% EL for nc 12 and 12% EL for nc 13 [13] (orange crosses with error bars in Fig 4C, see S1I Text).

### “*K*-or-more” model

Until now we assumed that the gene is read out only if all the binding sites are occupied. We relax this assumptions and consider the equilibrium “*K*-or-more” model (*P*_active_ ≡ *P*_i≥*K*_, 1 < *K* < *N*), where the gene is transcribed if at least *K* binding sites are occupied, assuming for simplicity that transcription occurs at the same rate regardless of the promoter state. As in the “all-or-nothing” model, the attainable pattern steepness is also bounded by the number of OS (*H* ≤ *N* − *τ*_bind_/*τ*_active_), but to achieve a specific steepness *H*, the *τ*_active_ in the “*K*-or-more” model is *N*−1 times smaller than that of “all-or-nothing” model. However, since the deactivation process now involves several reversible steps, *τ*_active_ is also noisier. As a result, the “*K*-or-more” model has only a slightly faster pattern formation time and slightly lower readout error than the “all-or-nothing” case (Fig N in S1 Text). In general, the ‘*K*-or-more” setup does not change the conclusions about the parameter regimes where the minimal value of the positional resolution Δ*X* can be obtained (Fig O in S1 Text).

### Transcription pattern formed by additional transcription factor gradients

We also investigated whether two mirrored transcription factor gradients, one anterior activator TF and one posterior repressor TF’, could lower the predicted pattern steepness, at the same time keeping low values of positional resolution. While there is no direct evidence for additional regulatory gradients acting in the early nuclear cycles, the idea of an inverse gradient, possibly indirectly due to Caudal, has been suggested [39]. We assume *N* = 6 binding sites for the Anterior-Posterior decreasing gradient (TF) and *L* = 6 binding sites for Posterior-Anterior decreasing (TF’) gradient. Transcription is allowed only when the promoter is fully bound by TF and free of TF’ and we assume that the interactions of TF and TF’ with the promoter are independent (see S1H Text). In the equilibrium model, the pattern can achieve a maximum steepness of H* ∼ 7 given the total of 12 binding sites (Fig 4D). The quantitative conclusions are the same as for the previously considered models (Figs P and Q in S1 Text) but the minimal value of the positional resolution (Δ*X* ∼ 10% EL in nc 12 and nc 13) is smaller than that achieved with a single TF gradient, and smaller than observed experimentally.

Lastly, we investigated the pattern formation when an additional repressor is concentrated in the mid-embryo region (see S1H Text). This scenario is motivated by the known pattern of the Capicua (Cic) protein and its potential effect on transcription. In the *hb* promoter sequence there is one known binding motif for the Cic protein [40]. Since the Cic concentration is relatively constant at the *hb* pattern boundary (∼−5% EL from mid-embryo), Cic does not affect the pattern steepness. We also find that the Cic gradient contributes little to the positional resolution of the *hb* pattern (Fig R in S1 Text).

### A common Hill coefficient for all nuclear cycles

**Fig 5:**
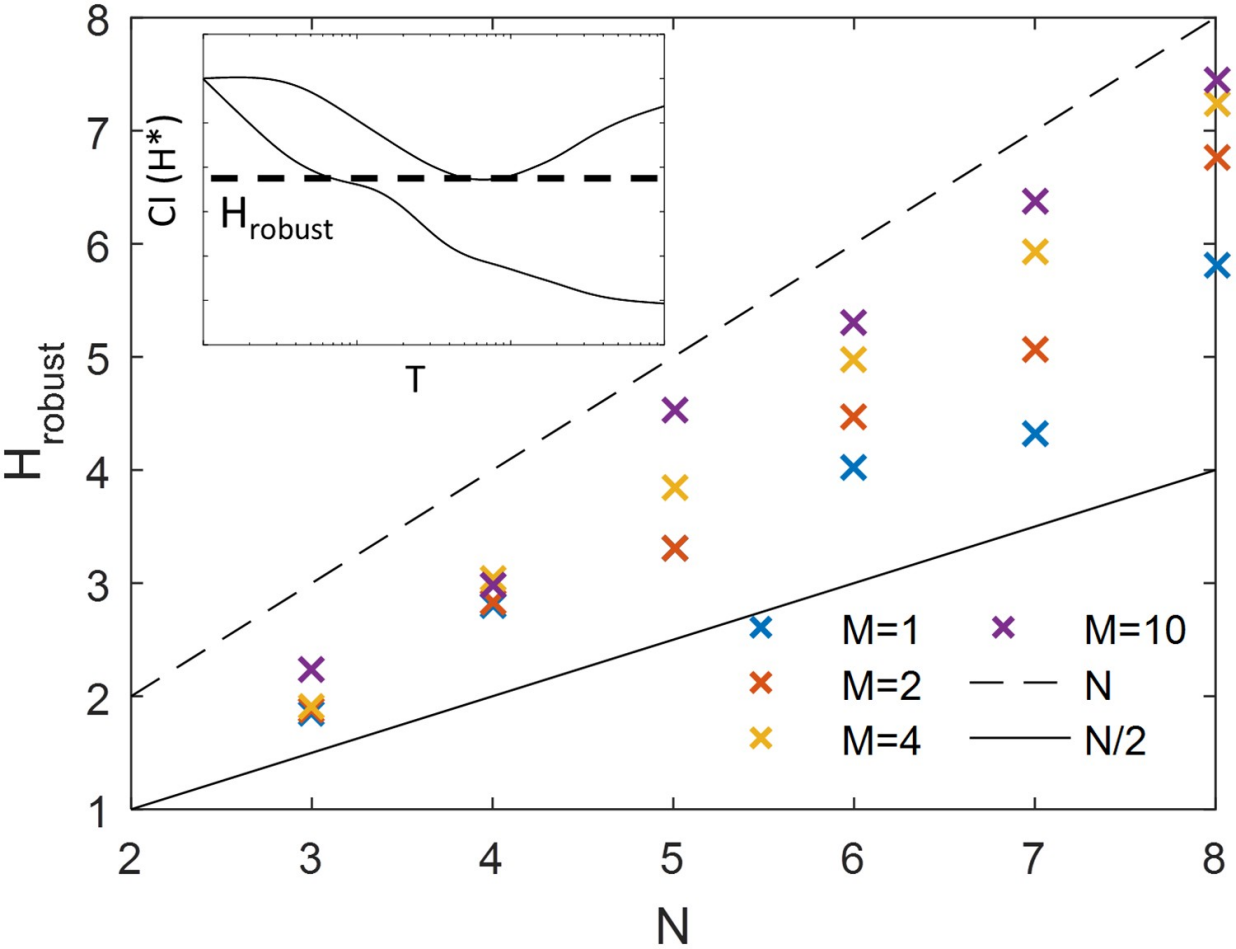
The Hill coefficient *H*_robust_ that gives the best positional resolution for the widest range of steady state transcription periods for the equilibrium model with *N* = 6 binding sites. Since the regulatory parameters are unlikely to change between nc 11-13, we look for the model quantified by its Hill coefficient that results in the lowest values of the positional resolution in the largest range of *T*. Inset: *H*_robust_ is calculated as the minimum of the upper bound of the confidence interval of the optimal Hill coefficient *CI*(H*), where the optimal Hill coefficient H* is defined in Fig 4A, and the confidence intervals are the solid blue lines in Fig 4B. *H*_robust_ is shown as a function of the OS number (*N*) and number of readout genes (*M*). Also plotted for reference are *H* = *N* (dashed line) and *H* = *N*/2 (solid line). *H*_robust_ for *M* = 1 is much less than the maximum steepness allowed by the model *H* = *N* and with a lot of spatial averaging (*M* = 10) *H*_robust_ approaches *N*.

Since the interphase duration varies during the early development phase but the molecular encoding of regulation is unlikely to change, we can use the results of the simplest equilibrium model Fig 4B to define a value of a Hill coefficient, *H*_robust_, that gives the minimal value of the positional resolution in the widest range of steady state transcription periods *T* (see Fig 5 inset) as a function of the number of operator sites (*N*) for different numbers of expressing gene copies (*M*). For *M* = 1, *H*_robust_ is slightly greater than *N*/2, resulting in not so steep boundaries (Fig 5). *H*_robust_ increases with *M* but is always smaller than its highest possible value of *N* allowed by the equilibrium model, even for very large numbers of expressing genes.

The optimal value of the Hill coefficients in nc 12 and 13 for all the considered models, as well as the *H*_robust_ values, are all between *H* ∼ 2 − 4. These values are in very good agreement with *in vitro* experiments that measured the cooperativity of 6 Bcd binding sites on the *hb* promoter [20, 41] (*H*_data_ ∼ 3).

### Comparison to experimental data

Comparing the model predictions to the experimental data [13], one can construct an equilibrium model that correctly captures the experimentally observed positional resolution, but it is much harder to achieve the readout steepness observed from the endogenous promoter given the currently identified number of binding sites. As has been shown before [18], non-equilibrium models allow for steeper expression profiles. However, increasing the Hill coefficients to *H*_data_ ∼ 7 [13, 16] also increases the minimal obtainable value of the positional resolution within a hybrid non-equilibrium model to Δ*X* ∼ 20% EL (∼10 nuclei widths), slightly above the the experimentally observed value of Δ*X* ∼ 12% EL in nc 13 (∼6 nuclei widths) (Fig 4C). Unfortunately, from the experimental data it is hard to reliably extract Hill coefficients for nc 11.

Steep boundaries are only possible if the promoter spends most of its time in the fully occupied or fully bound states, which sets boundaries on the switching parameters [30, 42] (Fig F in S1 Text). We looked for the kinetic parameter set that yields the smallest positional resolution Δ*X* and, although the values vary with the interphase duration, we find that a parameter set that results in the experimentally observed Δ*X*_data_ ∼ 12% EL in nc 12 does not change over multiple nuclear cycles of varying duration. This stability throughout the nuclear cycles is consistent with experimental observations that the Bcd interactions with the *hb* promoter are likely independent of other TF, which suggests the binding rate constant coefficients are independent of the nuclei’s positions along the AP axes [43].

Varying the only parameter of the model *τ*_bind_, which is set by the 3D diffusion assumption, rescales the steady state transcription period *T* (see Fig S in S1 Text). However, this rescaling does not quantitatively change the conclusions of our analysis for the equilibrium models, since only the non-equilibrium model with *N* = 6 binding sites is able to produce boundaries as steep as those observed in the experiments (Fig 4C). Within a non-equilibrium model longer binding timescales (*τ*_bind_ = 40*s*) than currently estimated within the diffusion approximation (*τ*_bind_ = 4*s*) result in a model that reproduces the observed steepness in nc 11-13 (Fig S in S1 Text) but, as discussed above, also results in a much higher minimal value of the positional resolution. Conversely, short binding timescales (*τ*_bind_ = 0.4*s*) allow the model to reach very low values of positional resolution in models with much smaller corresponding Hill coefficients than *H*_data_ (Fig S in S1 Text).

We also asked what value of the binding rate *τ*_bind_ in a non-equilibrium model results in both Hill coefficients and positional resolution that is consistent with experimentally observed values. For this, we calculate the positional resolution as a function of *τ*_bind_ and randomized the remaining set of binding and unbinding parameters to achieve the experimentally observed *H*_data_ ≈ 7 and the lowest value of the positional resolution given the fixed *H*_data_ constraint (see Fig V in S1 Text). The difference with the analysis in Fig S in S1 Text is that now we add an additional constraint on *H* = *H*_data_, so the minimal value of positional resolution is greater than in the results in Fig S in S1 Text. We find that for small values of *τ*_bind_ ∼ 0.01s, the mean values of the experimentally observed positional resolution (Δ*X*_data_ ≈ 14% EL in nc12 and Δ*X*_data_ ≈ 12% EL in nc13) are close to the minimal value calculated in the model (Fig V in S1 Text). Taking into account the confidence interval of the experimentally measured positional resolution, the experimental values are very close to the minimal predicted values of positional resolution even for *τ*_bind_ ∼ 0.1*s*. We conclude that a hybrid non-equilibrium model with *N* = 6 binding sites can reproduce both the experimentally observed Hill steepness and positional resolution, if the binding timescales are smaller than currently estimated.

Achieving small *τ*_bind_ ∼ 0.1–0.4*s* requires a diffusion coefficient of *D* ∼ 100*μm*^2^/*s*, which seems an order of magnitude larger than the current estimates (*D* ∼ 7.4*μm*^2^/*s*) [9, 33]. Misestimates in *τ*_bind_ = 1/*Dac*[TF]) coming from the binding site size *a* and Bcd concentration [*TF*] separately are unlikely to be at the origin of such a large difference. Even considering a combined effect of a misestimate in the binding site size, Bcd concentration and the diffusion coefficient, the diffusion coefficient would need to be an order of magnitude larger. However, a different diffusion model, such as a combination of a 1D and 3D TF search for the operator site [44] could help lower the binding timescale. As a result, a non-equilibrium model with a slight modification (additional binding site, additional regulation) and a smaller binding rate does seem a likely candidate for explaining the experimental data.

We can also compare the readout error *δmRNA*/〈*mRNA*〉 calculated directly from the MS2-MCP experiments in nc 12 and nc 13 (Fig U in S1 Text). The experimental readout error in nc 12 is *δmRNA*/〈*mRNA*〉 = 0.82 and in nc 13 is *δmRNA*/〈*mRNA*〉 = 0.69, which are lower than expected from the equilibrium model *CV*_*P*_ ∼ 1 for the maximum allowed Hill coefficient of *N* = 6, but higher than the *CV*_*P*_ ∼ 0.45 in nc 12 and *CV*_*P*_ ∼ 0.25 in nc 13 for the non-equilibrium hybrid model that yields the minimal value of the positional resolution. The higher experimentally observed readout error may be due to the the fact that the living embryo does not saturate the lower bound of positional resolution, as well as additional sources of noise in the experiments that are not considered in this model. These sources of noise include the random arrival times of RNA polymerases [45], non-uniform progression of the polymerases along the DNA [46] or additional modes of regulation that manifest themselves in bursty expression even in the anterior region where Bcd binding should be saturated [25, 47], and possibly experimental noise. To focus on the regulatory architecture, following previous work [48–52], we assumed the mean expression and noise at the promoter level is correlated with the mRNA readout. Exploring the role of these different sources of noise that lead to the observed readout error in conjunction with binding models of different complexity remains a future direction.

The *δmRNA*/〈*mRNA*〉 values reported above are also less than the previously reported *δmRNA*/〈*mRNA*〉∼1.5 [25] for nuclei in a 10% EL strip centered at mid-embryo for the same 4 nuclei in nc 13. In the previous analysis the embryos where aligned in the middle of the embryo (0% EL), which is close to the half-maximal expression point based on the mean probability of the nuclei to transcribe the gene at any point during the interphase. In the current analysis, based on the discussion in the experimental companion paper [13], we align the embryos at their half-maximal expression point of the integral fluorescence intensity, which is typically positioned anterior to the middle of the embryo at ∼−5% EL. These results suggest that either fluctuations in the Bicoid concentration between embryos influence *δmRNA*/〈*mRNA*〉, or that nuclei that are positioned to the posterior of the mid-boundary point (*X* = 0) contribute more to the readout error, which is likely due to their lower expression probability. We confirm the latter hypothesis by finding for the 4 embryos aligned at *X* = 0 in nc 13 the *CV*_*P*_ in a 5% strip around 0% EL, *CV*_*P*_ = 1.78, which is larger than the *CV*_*P*_ = 0.69 in the strip centered at −5% EL. However, without experiments that simultaneously measure Bcd concentrations and *hb* expression, we cannot rule out that fluctuations in the Bicoid concentration also play a role.

To explore the role of a transcriptional repressor in these trade-offs, we also considered the possibility of binding sites for an inversely directed gradient. The choice of a gradient repressor was arbitrary, since the only known mirror gradient in early fly development, Caudal, has no known binding sites in the *hb* promoter, no known repressor function in fly development and its maternal component has been shown to be non-essential in early fly development [53, 54]. Nevertheless it provided for a simple choice of parameters and was motivated by earlier theoretical ideas [39], and known activator-repressor pairs in other systems [55]. Since we are only looking at a small part of the embryo the precise form of the gradient will not strongly influence our qualitative conclusions, so we opted for the mirror image for simplicity. This two gradient model, even in its equilibrium version, does decrease the positional resolution in short cell cycles while increasing the steepness of the expression profile (Fig 4D). Again, the exact results of the model do not position the experimental results for the endogenous promoter within the predictions of the model, but for the two TF gradient the minimal value of the positional resolution observed at nc 12 is obtained at earlier nc with H* ∼ 7, Δ*X* ∼ 16% EL is not far from the experimentally measured value of Δ*X*_data_ ∼ 12% EL in nc 13 (Fig 4D). Together these results suggest that a repressor gradient working together with Bcd in a non-equilibrium setting, possibly with additional Bcd or Hb binding sites, could explain all of the experimentally observed results. Following the above results for different binding timescales (Fig V in S1 Text), an equilibrium repressor gradient model with a smaller *τ*_bind_ is another way to agree the model and the data.

There are also other repressor candidates in the fly development, such as Capicua, which is a known repressor gradient albeit with a different profile [56, 57]. For simplicity, motivated by Capicua, we studied a model with a constant additional repressor gradient in the middle of the embryo. Not surprisingly, due to its symmetry around the boundary, this type of gradient neither increases steepness nor severely modifies the readout error.

## Discussion

In order to better understand the trade-off between short cell cycles, steepness, readout error and positional resolution we studied a family of models where transcription is controlled by the binding and unbinding of the Bcd TF to multiple operator sites on the hb promoter: equilibrium binding models with different expression rules, non-equilibrium models and equilibrium models with two TF gradients.

One possible way to reconcile steep profiles with small values of positional resolution are additional unidentified binding sites in the promoter. Currently the minimal *hb* promoter used in the experiments we are analyzing [13] is known to consist of 6 Bcd binding sites, one proximal and one distal Hb binding site. Of course, it could also include unidentified binding sites. Since we were interested in nc 11-13—the early cell cycles when the profile is already steep—we did not include the Hb binding sites in our analysis. At that stage of development the zygotic Hb gradient is weak, although there exists a maternal step-like Hb profile with a smaller amplitude than the final zygotic profile [8]. Since these Hunchback gradients have the same direction as Bcd, Hb binding sites would most likely have the same effect as additional Bcd sites so we did not add them to the model promoter. However due to the step-like shape with a boundary in the middle of the emrbyo, maternal hunchback may play a role in establishing the steep profile. The usually characterized minimal *hb* promoter also includes one to two Zelda binding sites but they either do not change or they decrease the pattern steepness [13]. Nevertheless additional unknown Bcd binding sites would certainly increase steepness, as could Hb binding sites.

The disagreement between the model and the data is not manifested by the fact that the experimental points do not precisely fall on the theoretical predictions. The fly embryo does not need to function close to the optimal parameter regime and probably it does not. The disagreement arises because the experimentally measured values of these two observables, the Hill coefficient and the positional resolution, cannot be simultaneously obtained within the current regulatory model with the experimentally estimated diffusion limited binding time. In general, within the current models, steep boundaries increase the minimal obtainable value of the positional resolution. Specifically, the results of the model tell us that in the case of the observed steep profiles the best positional resolution that can be achieved has a much larger value than is experimentally measured. Since this is the minimal value of the positional resolution, the experimentally observed value of the positional resolution must be larger. Yet, in experiments we observe much smaller values of the positional resolution. This suggests different modes of regulation, such as described above, or smaller binding timescales than currently estimated (Fig V in S1 Text). Yet if this process is fast, and early fly development is very fast, the undiscovered modes of regulation have to be simple [43].

Another explanation to consider for the discrepancy between the experimental observations and our current discussion of the model is that the assumption we made about the positional error being minimized in the developing embryo is not valid. However, even if we relax this assumption, the general conclusions do not change: the Bcd-only equilibrium *N* = 6 model is not compatible with the experimentally observed Hill coefficient regardless of this assumption and in the hybrid non-equilibirum model, the predicted positional regulation for the experimentally observed Hill coefficient values within the current regulatory model is larger than the observed positional regulation (Fig M in S1 Text). Relaxing this assumption does, however, make it even more likely for models with different binding timescales or additional regulators or binding sites can explain both the observables simultaneously.

The observed steep boundaries minimize the positional resolution only for very short cell cycles. Another possible regulatory strategy involves setting up an imprecise boundary with low positional resolution at nc 11 using a steep expression profile. This boundary would further be refined during the following cell cycles, using additional regulatory mechanisms, such as Hb regulation or epigenetic modifications encoding memory in the translational state [9], leading to lower positional regulation. We also demonstrated that if the system starts from an out-of-steady-state condition after mitosis, the interphase duration may not be long enough for steep steady state expression patterns to establish (Fig C in S1 Text). This may lead the pattern to shift along the AP axis from nuclear cycle to nuclear cycle, as observed in fly development [9].

The “all or nothing” model is clearly a simplifying assumption but we have shown that a “*K*-or-more” model does not change the quantitative conclusions. In the “*K*-or-more” model, we further, incorrectly, assume that the transcription rate is the same for all of the promoter states that enable transcription. However, given the generality of our conclusions, introducing intermediate transcription rates would change the precise numerical values of the achievable positional resolution but not the general constraints on steepness and the positional resolution.

As has been pointed out in the context of maximizing information flow between the Bcd gradient and Hb output [38], very steep boundaries decrease the ability of the nuclei to discriminate between similar Bcd concentrations. The optimal expression profiles for minimizing positional resolution are always relatively steep *H* > 1, since large input fluctuations in the posterior end of the embryo coming from small Bcd concentrations limit extremely flat expression profiles. In general, we give a real biological example of the previously identified phenomenon that utlrasensitive systems require extremely slow receptor switching dynamics, which results in increased errors at the single-cell readout level [58]. Other trade-offs imposed by a need for a precise or informative readout have also been explored, including the energy—speed—accuracy constraint that shows that these three quantitates cannot be simultaneously optimized [59] or the cost of optimal information transmission in a finite time [60].

The variability in the expression states of different nuclei in the considered models comes from the binding and unbinding noise of TF to OS. The binding rates are assumed to be diffusion limited, which we implement using the Berg-Purcell bound [26]. In order to concentrate on the trade-off between steepness and positional resolution and simplify the parameter space exploration, we make the simplifying assumption that the binding and unbinding dynamics are uncoupled from diffusion. This approximation means that after an unbinding event the TF diffuses far enough from the OS so that it does not have an increased probability of binding compared to other TF molecules and its rebinding can be considered as an independent event [29]. For the equilibrium model, where all binding sites are the same, allowing for fast rebinding renormalizes the binding rates depending on the number of available free binding sites [29]. This renormalization would rescale the time axes to shorter times (or shift the time axis to the left on the log scale), but would not qualitatively change the discussed results (see Fig S in S1 Text). The effects of the full model of coupled binding and diffusion in the non-equilibrium model remain to be investigated in detail. Coupling the search process to the non-equilibrium process is also interesting in light of recent experimental evidence of two Bcd populations, one that spends a long time bound (∼ 1*s*)(< 0.1*s*) to the DNA, and the other that spends a short time bound (< 0.1*s*) [61], which could be a manifestation of specific or non-specific rebinding.

We compared the experimentally measured positional resolution and steepness in the MS2-MCP experiments [12, 13, 24] to the *M* = 1 “all or nothing” model, since these experiments look at heterozygous constructs. The developing fly embryo is homozygous and has M = 2 genes, and the total resolution of the gene readout that matters for downstream genes should be determined at the protein level. Therefore the overall resolution at the protein level is different than measured by the MS2-MCP system [15].

At the protein level Gregor *et al*. [15] measured a Hill coefficient of $H_{\text{data}}^{\text{protein}}=5$ in nc 14 and concluded that within the equilibrium limit of *H* ≤ *N* the known six binding sites are sufficient to achieve this steepness. In this work we consider the steepness of the *mRNA* readout in nc 13 and earlier, which is steeper (*H*_data_ ∼ 7) than the protein boundary at later cycles [13]. Our results therefore do not contradict previous observations [15]. The protein boundary is likely to benefit from averaging of protein concentrations between nuclei [15, 35]. The fast timescale of about 2 minutes for achieving the steep mRNA boundary [13] suggests that the readout mechanism initially produces a steeper boundary, which is then made less steep with time, possibly due to diffusion [35]. While spatial averaging is clearly important for Hb proteins [15, 35], given that the steep expression profile is established in ∼2 minutes [13], spatial averaging of *hb* mRNA in nc 11-12 probably plays a smaller role.

Inspired by the experiments of Lucas *et al*. [13] we focused on nc 11-13. The *hb* gene is also expressed during later stages of development [62–64]. In nc 14, additionally to the proximal promoter active in nc 11-13, expression of *hb* is also controlled by distal and shadow enhancers [1, 2, 43]. However they are unlikely to play a major role in nc 11-13. Recent studies have also used an optogenetically modifiable Bcd protein [65] that makes it possible to modify the transcription of Bcd target genes. Combining all these experimental approaches with the knowledge gained both about *hb* mRNA [12, 13, 24] and Hb protein regulation [36] is a much needed future direction.

In summary, we show how trade-offs between steep expression profiles and positional resolution influence the possible regulatory modes of *hb* expression in the short early cell cycles of fly development. We propose a number of possible solutions from non-equilibrium binding, additional regulatory gradients and binding sites, faster binding rates to epigenetic regulation. Additional experiments are needed to discriminate between the proposed scenarios. For example, testing whether the binding of TF to the promoter is equilibrium or non-equilibrium requires analysis of experiments that track TF bound to fluorescent probes that follow their binding and unbinding. Equilibrium dynamics results in time reversible traces—a property that can be evaluated based on such tagged TF data collected using high resolution microscopy.

## Methods

### Model of promoter dynamics

The general model of transcription regulation through transcription factor (TF) binding/unbinding to the operator sites (OS) is based on the graph-based framework of biochemical systems [18, 66]. In short, for a promoter with *N* TF binding sites the model considers all the possible 2^*N*^ promoter occupancy states and all transitions between these states that involve the binding and unbinding of one TF. In most treatments of the model we randomize parameters to explore its behavior. The full non-equilibrium model is described in S1A Text and solved numerically. Assuming the binding sites are indistinguishable results in the one dimensional equilibrium model in Eq 2.

### Randomization of kinetic parameters

The kinetic rate constants are randomized in $R^{+}$ space. Assuming binding is diffusion limited by the Berg-Purcell limit [26], the binding rate constants *k*_*i*_ have an upper bound depending on the OS search time *τ*_bind_. Based on measured and typically taken parameters for diffusion, concentration and operator size the we estimate *τ*_bind_ = 4*s* (S1A Text). However, since we randomize the parameters, our quantitative conclusions do not depend on the exact values taken for these parameters. For the non-equilibrium model, *i* ranges from 1 to 2^*N*−1^ and there are no further constraints on the binding rates. For the equilibrium model, a reaction from *P*_*i*−1_ to *P*_*i*_ is the binding of a TF to one of the remaining *N* − *i* + 1 free OS, so the rate constants *k*_+*i*_ are bound by N − i + 1)/*τ*_bind_. There are no bounds on the unbinding rate constants *k*_−*i*_, but their values are rescaled *a posteriori* so that the boundary is located in the middle of the embryo (*P*(*P*_*active*_, *X* = 0) = 0.5, see S1B Text). The values of the rate constants are sampled to be uniformly distributed on the logarithmic scale, from 10^−20^ *s*^−1^ to 10^20^ *s*^−1^. The number of randomized configurations tested is on the order of 10^5^.

### Calculating the positional resolution

To find the value of Δ*X* for a specific kinetic parameter set, we test the condition *P*(*F*_+_ ≤ *F*_−_) ≤ 0.05 with increasing nuclei distance Δ*W*. The distribution of *F*_+_ and *F*_−_ is taken as the marginal distributions of the gene readout from 500 stochastic simulation runs (SSA) [67, 68] implemented in the SGNS2 simulator [69]. *F*_−_ and *F*_+_ are not well-fit by Gaussian distributions, especially for short interphase durations. Δ*X* is the smallest value of Δ*W* yielding a tolerable error of *P*(*F*_+_ ≤ *F*_−_) ≤ 0.05. Δ*X* and Δ*W* and nuclei position *X* can be expressed in units of length relative to the decay length of the TF gradient *λ* ≈ 100*μm* [5], which corresponds to ∼20% of the embryo length (EL).

### Experimental data

The data on the dynamics of *hb* pattern are taken from Lucas *et al*. 2018 [13]. In this work, *hb* transcription in nuclear cycle 11 to 13 is monitored using the MS2-MCP RNA tagging system [12, 14]. From the total amount of mRNA produced per nuclei at any given position, we extracted the pattern steepness (*H*_data_) and positional resolution (Δ*X*_data_) (see S1I Text).

## Supporting information

S1 Text (PDF)
