# Supplementary material for: Precision in a rush: Trade-offs between reproducibility and steepness of the hunchback expression pattern
Source: PLoS Comput Biol. 2018 Oct 11;14(10):e1006513. doi: 10.1371/journal.pcbi.1006513 (PMC6198997; doi:10.1371/journal.pcbi.1006513)
Supplement: S1 Text — (PDF) [file pcbi.1006513.s001.pdf]

Precision in a rush: trade-offs between reproducibility and steepness of the hunchback expression pattern

Supporting Information - Figures only (for referencing purpose)

October 1, 2018

A

Model of transcription regulation

The binding site model

We use a model of transcription factors (TF) binding/unbinding to operator sites (OS) based on the graph-based method introduced in [1] and implemented in [2]. Within this framework one considers a finite, connected, labeled, directed graph $[\overline{v}, \overline{e}]$, where the vertices $\overline{v}$ describe the promoter micro-states corresponding to the number and arrangement of bound TF on the promoter OS array, and the edges $\overline{e}$ are transitions between micro-states. The edge labels $\overline{k}$ are infinitesimal transition rates for a Markov process.

For a gene whose promoter has N OS, the number of micro-states/vertices is 2^N:

$$\overline{v} = [v_1, v_2...v_{2^N}]. \tag{1}$$

Since we assume only one binding/unbinding event can take place at a time, not all vertices are directly connected and the number of edges is $N_{\text{edges}} = N2^N$ [2].

We separate the edges $\overline{e}$ into two sets, corresponding to forward (binding) transitions (e_+) and backward (unbinding) transitions (e_-):

$$e = [e_+, e_-] = [e_{+1}, e_{+2}, ...e_{+N2^{N-1}}, e_{-1}, e_{-2}, ...e_{-N2^{N-1}}], \tag{2}$$

with the corresponding edge labels describing the reaction rate constants of binding and unbinding between the TF and the OS:

$$\overline{k} = [k_+, k_-] = [k_{+1}, k_{+2}, ...k_{+N2^{N-1}}, k_{-1}, k_{-2}, ...k_{-N2^{N-1}}]. \tag{3}$$

An example of the labeled graph is shown in Figure A for $N = 3$.

The micro states of the gene are divided into active (when the gene is expressed) and inactive (when the gene is not expressed) states. In this work, we assume that the gene is activated only when the OS are bound by at least K TFs (corresponding to the "K-or-nothing" case in [2], with $K \leq N$). During this active state window RNA polymerases can bind to the target promoter to initiate transcription with a rate that is much faster than the rate of gene activation. Therefore, the mean transcription rate and the mean expression values of a gene can be approximated by the probability of the gene to be active $\langle f(X) \rangle = P(P_{i \geq K})$.

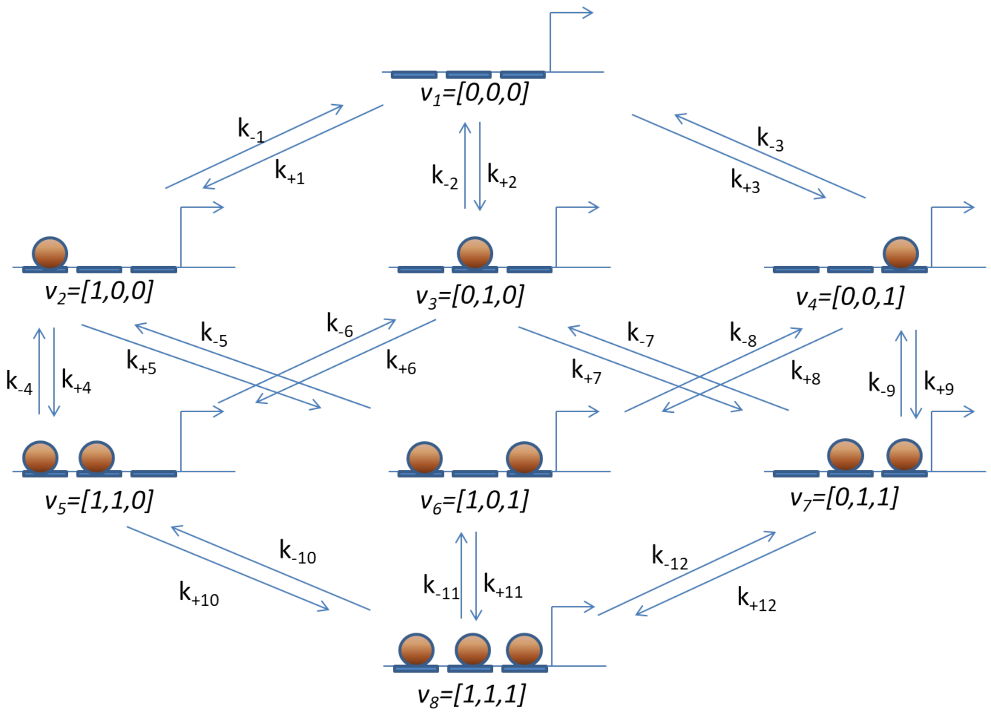

Figure A: **The labeled graph for the general non-equilibrium binding site model of transcription regulation for $N = 3$ OS**. Each vertex v corresponds to a unique OS array state. The edge labels describe the binding and unbinding reactions, assuming one reaction takes place at a time.

The thermodynamic equilibrium model

One dimensional model

We assume that the N binding sites are identical, and thus all the micro-states $v = [v_i]_{i=1..N}$ are characterized only by the number of bound TF (Σv_i). In this case the model is reduced to a one dimensional model described by P_i – promoter states with i bound TF molecules:

$$P_0 \underset{k_{-1}}{\overset{k_1[TF]}{\rightleftharpoons}} P_1 \underset{k_{-2}}{\overset{k_2[TF]}{\rightleftharpoons}} P_2 ... \underset{k_{-N}}{\overset{k_N[TF]}{\rightleftharpoons}} P_N, \tag{4}$$

where the TF-OS interactions are at thermodynamic equilibrium and the detailed balance is satisfied. The binding and unbinding of Bcd molecules to the OS occurs with rate constants k_i and k_{-i}. The maximum value of k_i is dependent on τ_{bind} the time for a free OS to be bound by TF and the number of free remaining operator $[N - i + 1]$: $k_i \leq [N - i + 1]/\tau_{\text{bind}}$. $[TF]$ is the normalized TF concentration, and is equal to 1 at the mid-boundary position $X = 0$.

Steady-state solution

The temporal evolution of the probability $P(P_i)$ that the promoter is in state i in the one dimensional model in Eq. 4 is given by:

$$\partial_t P(P_i) = k_i[TF]P(P_{i-1}) + k_{-(i+1)}P(P_{i+1}) - (k_{-i} + k_{i+1}[TF])\, P(P_i). \tag{5}$$

The steady state solution is:

$$P(P_i, [TF]) = \frac{\prod_{j=0}^{i} K_j [TF]^i}{\sum_{\ell=0}^{N} \prod_{j=0}^{\ell} K_j [TF]^\ell}, \tag{6}$$

where $K_j = k_j/k_{-j}$ are the equilibrium constants for each transition between two states, and $K_0 = 1$.

We introduce the effective equilibrium constant $\tilde{K}_i = \prod_{j=0}^{i} K_j$ that is proportional to the fraction of time the promoter spends in state P_i:

$$P(P_i, [TF]) = \frac{\tilde{K}_i [TF]^i}{\sum_{j=0}^{N} \tilde{K}_j [TF]^j}. \tag{7}$$

If the TF concentration gradient follows an exponential curve, we can express the TF concentration $[TF]$ in terms of the nuclei's position (Eq. 14) and Eq. 7 becomes:

$$P(P_i, X) = \frac{\tilde{K}_i e^{iX}}{\sum_{j=0}^{N} \tilde{K}_j e^{jX}}. \tag{8}$$

Promoter searching time at the boundary position

We estimate the expected time for a binding event between a single binding site and a TF at the mid-boundary position ($[TF] = 1$).

Assuming that the TF can only search for OS by diffusing in the nucleus and that each collision between TF and OS is one successful binding event [3], we estimate $\tau_{bind} = 1/(Dac) \sim 4$ s, using $D \sim 7.4 \mu m^2/s$ – the diffusion coefficient of TF (measured through Bcd-eGFP using FRAP [4, 5]), $[c \sim 11.2/\mu m^3$ [4] – the absolute TF concentration at the mid-boundary position and $a \sim 3nm$ [6] – the size of one operator site for Bicoid.

B

Aligning the pattern boundary position

From the randomized kinetic parameter set $\overline{k} = [k_i, k_{-i}]$, we solve the probability of the gene to be active at any position position X and find the mid-boundary position $X_0(\overline{k})$ such that $P(P_{\text{active}}|\overline{k}, X_0(\overline{k})) = 0.5$. The solution remains the same when we multiply both k_i and k_{-i} to a factor of $e^{X_0(\overline{k})}$:

$$
\begin{aligned}
P(P_{\text{active}}|\overline{k}, X) &= P(P_{active}|[k_i, k_{-i}], X) && (9)\\
&= P(P_{\text{active}}|[k_i e^{X_0((\overline{k}))}, k_{-i} e^{X_0((\overline{k}))}], X) && (10)\\
&= P(P_{\text{active}}|[k_i, k_{-i} e^{X_0((\overline{k}))}], X - X_0(\overline{k})). && (11)
\end{aligned}
$$

The whole pattern can be shifted so that the mid-boundary position is located at $X_0(\overline{k}) = X = 0$:

$$
P(P_{\text{active}}([k_i, k_{-i} e^{X_0(\overline{k})}], 0) = P(P_{active}|[k_i, k_{-i}], X_0(\overline{k})) = 0.5. \quad (12)
$$

We obtain the new parameter set $\overline{k'} = [k_i, k_{-i} e^{X_0(\overline{k})}]$, which satisfies the model assumptions $X_0(\overline{k'}) = 0$.

C

Boundary steepness and the Hill coefficient

The boundary steepness is a feature that emerges only when looking at the mean transcription readout value $f_P(X)$ of the hunchback gene along the AP axis (see Fig. 1 C and D of the main text) [2, 7, 8, 6]. We assume that the mean expression of the *hunchback* gene is regulated by a single transcription factor (i.e. Bcd), which has normalized concentration [TF], in terms of a Hill function with coefficient H:

$$\langle f_P([TF]) \rangle = \frac{[TF]^H}{1 + [TF]^H}. \tag{13}$$

In the case of Bcd-*hb* system the TF concentration decays exponentially along the embryo length [9] and we can estimate the nuclei's position X from the TF concentration:

$$X = \ln([TF]). \tag{14}$$

X is reported in units of decay length λ of the TF gradient, which is $\sim 100\mu m$ or 20% of the embryo length (EL) [9]. Eq. 13 becomes:

$$\langle f_P(X) \rangle = \frac{e^{H \cdot X}}{1 + e^{H \cdot X}}. \tag{15}$$

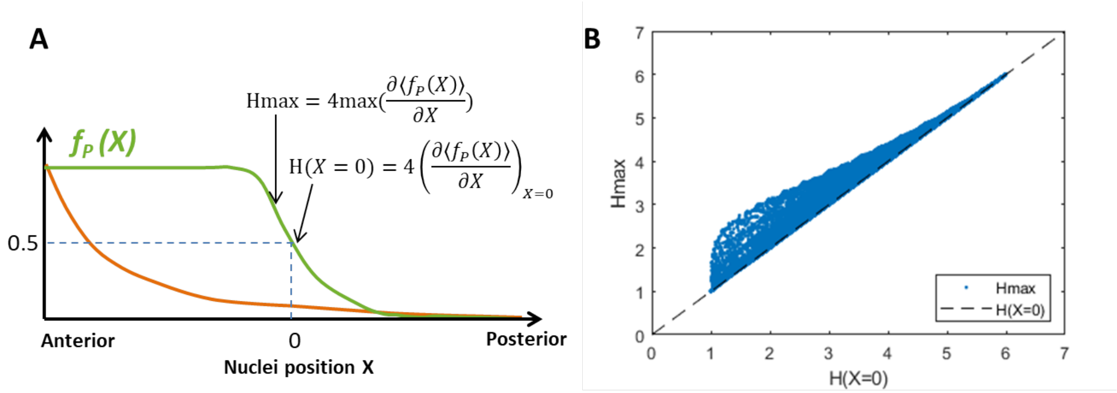

Figure B: **Quantifying the pattern steepness**.
Panel A: From the mean readout function $f_P(X)$, the Hill coefficient H can be obtained from either the slope at the mid-boundary position corresponding to half-maximum readout of $\langle f_P(X) \rangle$, $(H(X = 0))$ or at the steepest point $(H_{\max})$ as in [2].
Panel B: Comparison between the two definitions of steepness $H(X = 0)$ and $H_{\max}$ for the equilibrium regulatory model with $N = 6$ binding sites shows the two values are correlated. The data points are taken from ~ 50000 data points with randomized kinetic parameters.

We define the mid-boundary position, $X = 0$, as the position along the AP axis corresponding to half-maximum of the mean readout function, $\langle f_P(X) \rangle = 0.5$. Note that the expression boundary is not necessarily positioned at the middle of the embryo.

Hill coefficients are typically obtained either by fitting the mean expression function to a sigmoid curve [6, 8, 7] or by comparing the maximum derivative of the mean readout function to that of

a sigmoid function [2]. Here, to easily compare different embryos to each other and to analytical predictions, we calculate the Hill coefficient by comparing the slope of the mean readout function at the mid-boundary position ($X = 0$) to the prediction of Eq. 15:

$$H = 4\big(\frac{\partial\langle f_P(X)\rangle}{\partial X}\big)_{X=0}. \tag{16}$$

To see if our definition using the derivative at the half-maximum expression position significantly changes the numerical value of the steepness when calculated at the point of the maximum derivative (Figure B Panel A), we compare the two values obtained at the steady state of the transcription regulatory model defined in A for different sets of randomized kinetic parameters. The results shown in Figure B Panel B for $N = 6$ show the two values of the steepness calculated at different positions are tightly correlated, especially in the regime of high steepness. For the remainder of this work, we work with the steepness defined at mid-boundary ($H(X = 0)$) and note that an alternative definition would not change our conclusions.

D

Boundary steepness and promoter switching time for the equilibrium model

The boundary steepness

We consider the general "K-or-more case", that is the promoter is active when at least K OS are bound by TF, $P(P_{\text{active}}) = \sum_{i=K}^{N} P(P_i)$. When $K = N$, we recover the "all-or-nothing" case, $P(P_{\text{active}}) = P(P_N)$.

At the boundary position $X = 0$ and $P(P_{\text{active}}) = p$ ($0 \leq p \leq 1$), Eq. 7 simplifies to:

$$P(P_{\text{active}}, X = 0) = \frac{\sum_{i=K}^{N} \tilde{K}_i}{\sum_{j=0}^{N} \tilde{K}_j} = p, \tag{17}$$

which imposes a condition on the effective equilibrium constants:

$$\sum_{i=K}^{N} \tilde{K}_i = \frac{p}{1-p} \sum_{k=0}^{K-1} \tilde{K}_k = p \sum_{j=0}^{N} \tilde{K}_j. \tag{18}$$

The slope of the pattern at mid-boundary position is given by the derivative:

$$\begin{aligned}
\left(\frac{\partial P(P_{\text{active}})}{\partial X}\right)\Big|_{X=0} &= \sum_{i=K}^{N} \left(\frac{\partial}{\partial X} \frac{\tilde{K}_i e^{iX}}{\sum_{j=0}^{N} \tilde{K}_j e^{jX}} \right)\Big|_{X=0} \\
&= \frac{\sum_{i=K}^{N} i\tilde{K}_i}{\sum_{j=0}^{N} \tilde{K}_j} - \frac{\sum_{i=K}^{N} \tilde{K}_i \sum_{j=0}^{N} j\tilde{K}_j}{(\sum_{j=0}^{N} \tilde{K}_j)^2} \\
&= \frac{\sum_{i=K}^{N} i\tilde{K}_i}{\sum_{j=0}^{N} \tilde{K}_j} - \frac{p\sum_{j=0}^{N} j\tilde{K}_j}{\sum_{j=0}^{N} \tilde{K}_j},
\end{aligned} \tag{19}$$

where in the last step we used Eq. 18.

For clarity, we set the ranges $i = K..N$, $j = 0..N$ and $k = 0..K-1$. Eq. 19 is then rewritten as:

$$\begin{aligned}
\left(\frac{\partial P(P_{\text{active}})}{\partial X}\right)_{X=0} &= \frac{\sum i\tilde{K}_i - p\sum j\tilde{K}_j}{\sum \tilde{K}_j} \\
&= \frac{(1-p)\sum i\tilde{K}_i - p\sum k\tilde{K}_k}{\sum \tilde{K}_j} \\
&= \frac{N(1-p)\sum \tilde{K}_i}{\sum \tilde{K}_j} - \frac{(1-p)\sum (N-i)\tilde{K}_i + p\sum k\tilde{K}_k}{\sum \tilde{K}_j} \\
&= Np(1-p) - \frac{(1-p)\sum (N-i)\tilde{K}_i + p\sum k\tilde{K}_k}{\sum \tilde{K}_j},
\end{aligned} \tag{20}$$

and the Hill coefficient (Eq. 16) is:

$$H = 4\frac{\partial P(P_{\text{active}})}{\partial X}$$
$$= 4Np(1-p) - 4\frac{(1-p)\sum(N-i)\tilde{K}_i + p\sum k\tilde{K}_k}{\sum \tilde{K}_j}. \quad (21)$$

At the boundary criteria $p = 0.5$ and:

$$H = N - 2\frac{\sum(N-i)\tilde{K}_i + \sum k\tilde{K}_k}{\sum \tilde{K}_j}. \quad (22)$$

In the "all-or-nothing" case ($K = N$), $\sum_{j=0}^{N} \tilde{K}_j = 2\tilde{K}_N$ (Eq. 18), the first term in the nominator disappears and Eq. 22 becomes

$$H = N - \frac{\sum_{k=0}^{N-1} k\tilde{K}_k}{\tilde{K}_N}. \quad (23)$$

Bounds for pattern steepness

Eq. 22 gives an upper bound of $H \approx N$ at the mid-boundary position, which occurs when

$$\frac{\sum_{i=K}^{N}(N-i)\tilde{K}_i + \sum_{k=0}^{K-1} k\tilde{K}_k}{\sum_{j=0}^{N} \tilde{K}_j/2} << N. \quad (24)$$

When N is not too large (≤ 10), we can rewrite the upper bound condition in Eq. 24:

$$\sum_{i=K}^{N}(N-i)\tilde{K}_i + \sum_{k=0}^{K-1} k\tilde{K}_k \ll \sum_{j=0}^{N} \tilde{K}_j/2 \quad (25)$$

which is equivalent to $\tilde{K}_l \ll \tilde{K}_0 + \tilde{K}_N$ for $l = 1..N-1$ or $\tilde{K}_0 + \tilde{K}_N \approx \sum_{j=0}^{N} \tilde{K}_j$.

Maximum sharpness ($H = N$) is achieved when $\tilde{K}_0 \approx \tilde{K}_N \approx 0.5\sum_{i=0}^{N} \tilde{K}_i$ – the system spends most of the time in the fully free (P_0) or fully bound states (P_N). In this limit, we have $P(P_0) + P(P_N) \approx 1$, and thus $P(P_{active}) \approx P(P_N)$ regardless of the value of K.

To find the lower bound on H, we consider the difference between H and N from Eq. 22:

$$N - H = \frac{\sum_{i=K}^{N}(N-i)\tilde{K}_i + \sum_{k=0}^{K-1} k\tilde{K}_k}{\sum_{j=0}^{N} \tilde{K}_j/2} \quad (26)$$
$$= \frac{\sum_{i=K}^{N}(N-K)\tilde{K}_i - \sum_{i=K}^{N}(i-K)\tilde{K}_i}{\sum_{i=K}^{N} \tilde{K}_i} + \frac{\sum_{k=0}^{K-1}(K-1)\tilde{K}_k - \sum_{k=0}^{K-1}(K-1-k)\tilde{K}_k}{\sum_{k=0}^{K-1} \tilde{K}_k}$$
$$= N - K - \frac{\sum_{i=K}^{N}(i-K)\tilde{K}_i}{\sum_{i=K}^{N} \tilde{K}_i} + K - 1 - \frac{\sum_{k=0}^{K-1}(K-1-k)\tilde{K}_k}{\sum_{k=0}^{K-1} \tilde{K}_k},$$
$$\leq N - 1$$

Thus $H \geq 1$. $H = 1$ when the sum in Eq. 25 are negligible compared to 1, which happens when $\tilde{K}_K \gg \tilde{K}_{K+1..N}$ and $\tilde{K}_{K-1} \gg \tilde{K}_{0..K-2}$. With these conditions, the promoter spends most of the time in P_{K-1} and P_K.

Promoter activity time

τ_{active} is the mean duration the promoter is in the active state and the system is at steady state, $\tau_{\text{active}} \sim P(P_{\text{active}})$. We can relate τ_{active} to the average time τ_N the promoter spends in the P_N state where all the operator sites are occupied by TF:

$$\begin{aligned}\tau_{\text{active}} &= \tau_N \frac{P(P_{\text{active}})}{P(P_N)} \\ &= \frac{1}{k_{-N}} \frac{\sum_{i=K}^{N} \tilde{K}_i}{\tilde{K}_N} \\ &= \frac{K_N}{k_N} \frac{\sum_{i=K}^{N} \tilde{K}_i}{\tilde{K}_N} \\ &= \frac{1}{k_N} \frac{\tilde{K}_N}{\tilde{K}_{N-1}} \frac{\sum_{i=K}^{N} \tilde{K}_i}{\tilde{K}_N} \\ &= \tau_{\text{bind}} \frac{\sum_{j=0}^{N} \tilde{K}_j}{2\tilde{K}_{N-1}},\end{aligned} \tag{27}$$

where $\tau_{\text{bind}} = 1/k_N$ is the expected time for a binding event between the remaining free OS of P_{N-1} and a TF at the mid-boundary position ($[TF] = 1$) (as defined in A).

Eq. 27 allows us to connect the Hill coefficient in Eq. 23 to τ_{active}. For $K = N$ (the "all-or-nothing" case), using Eq. 27, Eq. 23 becomes:

$$\begin{aligned}H &= N - \frac{\sum_{k=0}^{N-1} k\tilde{K}_k}{\sum_{j=0}^{N} \tilde{K}_j/2} \\ &= N - \frac{(N-1)\tilde{K}_{N-1} + \sum_{k=0}^{N-2} k\tilde{K}_k}{\sum_{j=0}^{N} \tilde{K}_j/2} \\ &= N - \frac{(N-1)\tau_{\text{bind}}}{\tau_{\text{active}}} - \frac{\sum_{k=0}^{N-2} k\tilde{K}_k}{\sum_{j=0}^{N} \tilde{K}_j/2} \\ &\leq N - \frac{(N-1)\tau_{\text{bind}}}{\tau_{\text{active}}},\end{aligned} \tag{28}$$

or

$$\tau_{\text{active}} \geq \tau_{\text{bind}} \frac{N-1}{N-H}. \tag{29}$$

This leads to the bound on the Hill coefficient presented in the main text:

$$H \leq N - (N-1)\frac{\tau_{\text{bind}}}{\tau_{\text{active}}}. \tag{30}$$

Given the estimate $\tau_{\text{bind}} \approx 4$s (Section A), $H \sim N$ for $\tau_{\text{active}} \gg 4$ s or $k_{-N} \ll 0.25\ s^{-1}$.

For $K < N$ (the "K-or-more" case), Eq. 22 becomes:

$$\begin{aligned}H &= N - \frac{\tilde{K}_{N-1} + \sum_{m=K}^{N-2}(N-m)\tilde{K}_m + \sum_{k=0}^{K-1} k\tilde{K}_k}{\sum_{j=0}^{N} \tilde{K}_j/2} \\ &= N - \frac{\tau_{\text{bind}}}{\tau_{\text{active}}} - \frac{\sum_{m=K}^{N-2}(N-m)\tilde{K}_m + \sum_{k=0}^{K-1} k\tilde{K}_k}{\sum_{j=0}^{N} \tilde{K}_j/2} \\ &\leq N - \frac{\tau_{\text{bind}}}{\tau_{\text{active}}},\end{aligned} \tag{31}$$

or

$$\tau_{\text{active}} \geq \tau_{\text{bind}} \frac{1}{N - H}. \tag{32}$$

The equality in Eq. 28 and Eq. 31 occurs when $\tilde{K}_{1..N-2} \ll \sum_{j=0}^{N} \tilde{K}_j$ – the system spends most of the time in the P_0, P_{N-1} and P_N states.

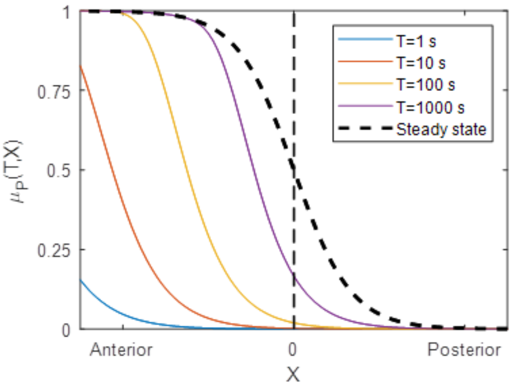

Figure C: **Example of the pattern formation following mitosis**. The mean promoter activity pattern $\mu_P(T, X)$ as a function of the position in the embryo the end of an interphase of duration T (solid colored lines), for interphases of varying duration. The dashed line shows the steady-state expression pattern.

E

Calculating the mean promoter activity and readout error

In this section we obtain analytical solutions for the time dependent mean promoter activity ($\mu_P(T, 0)$) and readout error ($CV_P(T)$). Those results are expressed in terms of the exponential of the transition rate matrix U of size $N2^N$ for the non-equilibrium model and size $N + 1$ for the equilibrium model, defined in Eq. 5. We discuss in what cases the matrix exponentiation can be done analytically or must be done numerically.

The steady state solution for the promoter activity probability vector is given by $U\overline{x} = 0$ and the normalization condition $\sum \overline{x} = 1$. In the equilibrium model, the steady state solution $\overline{x}$ is given by Eq. 8.

Mean promoter activity out of steady state

We define x_0 as the promoter state probability at the beginning of the interphase ($\sum x_0 = 1$). The mean promoter activity level at time T is given by:

$$\mu_P(T) = \alpha^{\mathrm{T}} e^{U \cdot T} x_0 \tag{33}$$

where α is a vector of the promoter active states. The ith element of α takes values of either 1 or 0, indicating the ith promoter state is active or inactive respectively.

The readout error

After the interphase of duration T, we obtain a readout $f_P(T)$ which is the average of the promoter activity trace $n(t)$ over time T:

$$f_P(T) = \frac{1}{T} \int_0^T n(t) dt. \tag{34}$$

At steady state ($x_0 = \overline{x}$), the probability of the gene to be active is a projection of the steady state probability onto the state of the system:

$$\langle f_P \rangle = \alpha^{\mathrm{T}} \times \overline{x}. \tag{35}$$

Let us define x_{fire} in which $x_{\text{fire}}(i) = \alpha(i)\overline{x}(i)$. The second moment of $f_P(T)$ can be found via the autocorrelation function:

$$\begin{aligned}\langle f_P^2(T) \rangle &= \frac{1}{T^2}\int_0^T du \int_0^T dv\ \alpha^{\mathrm{T}} e^{U|u-v|} x_{\text{fire}} \\ &= \frac{2}{T^2}\int_0^T du \int_0^u dv\ \alpha^{\mathrm{T}} e^{U(u-v)} x_{\text{fire}} \\ &= \frac{2\alpha^{\mathrm{T}}}{T^2}\int_0^T ds \int_s^T du\ e^{Us} x_{\text{fire}} \\ &= \frac{2\alpha^{\mathrm{T}}}{T^2}\left[\int_0^T ds (T-s) e^{Us}\right] x_{\text{fire}}.\end{aligned} \tag{36}$$

We diagonalize the matrix $U = VDV^{-1}$, where V is the eigenvector matrix and D a diagonal matrix of eigenvalues $[\lambda_1, \lambda_2, ...\lambda_M]$. Eq. 36 becomes

$$\langle f_P^2(T) \rangle = \frac{2\alpha^{\mathrm{T}}}{T^2} V \cdot \mathrm{diag}\big[L_1, L_2, ...L_M\big] \cdot V^{-1} x_{\text{fire}} \tag{37}$$

with

$$L_i = \int_0^T ds (T-s) e^{\lambda_i s}. \tag{38}$$

Performing the integration, for $\lambda_i = 0$:

$$L_i = \int_0^T ds (T-s) = T^2 - T^2/2 = T^2/2, \tag{39}$$

and for $\lambda_i \neq 0$:

$$\begin{aligned}L_i &= \int_0^T ds \cdot T \cdot e^{\lambda_i s} - \int_0^T ds \cdot s \cdot e^{\lambda_i s} \\ &= \int_0^T ds \cdot T e^{\lambda_i s} - 1/\lambda_i \big(s \cdot e^{\lambda_i s}\big|_0^T + \int_0^T ds \cdot T \cdot e^{\lambda_i s}\big) \\ &= T/\lambda_i (e^{\lambda_i T} - 1) - T/\lambda_i e^{\lambda_i T} + 1/\lambda_i^2 (e^{\lambda_i T} - 1) \\ &= 1/\lambda_i^2 (e^{\lambda_i T} - 1) - T/\lambda_i.\end{aligned} \tag{40}$$

The readout error $CV_P(T)$ is calculated as:

$$CV_P(T) = \frac{\delta f_P(T)}{\langle f_P \rangle} = \sqrt{\frac{\langle f_P^2(T) \rangle - \langle f_P \rangle^2}{\langle f_P \rangle^2}} \tag{41}$$

In the special case when H approaches its maximum value, τ_{active} is infinitely long, all eigenvalues λ_i become zero and $L_i = T^2/2$ for all i. In this limiting case:

$$\begin{aligned}\langle f_P^2(T) \rangle &= \frac{2\alpha^{\mathrm{T}}}{T^2} V \cdot T^2/2 \cdot V^{-1} \overline{x} \\ &= \alpha^{\mathrm{T}} \overline{x} \\ &= \langle f_P \rangle\end{aligned} \tag{42}$$

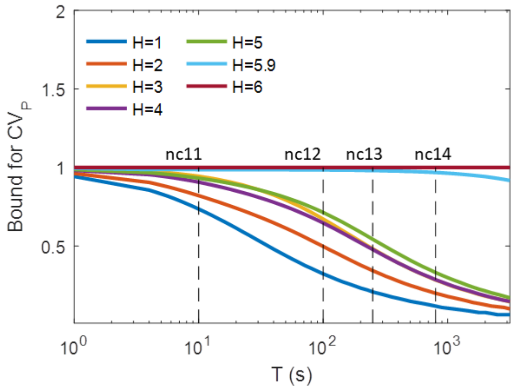

Figure D: **The readout error of [TF] readout at steady-state.** The smallest values of CV_P as a function of interphase duration T, plotted for varying pattern sharpness H.

Applying Eq. 42 to Eq. 41, the readout error at the mid-boundary position is therefore:

$$CV_P(T) = \sqrt{\frac{\langle f_P \rangle - \langle f_P \rangle^2}{\langle f_P \rangle^2}} = 1 \tag{43}$$

Specific case with 2 binding sites

In the specific case $N = 2$:

$$P_0 \underset{k_{-1}}{\overset{k_1[TF]}{\rightleftharpoons}} P_1 \underset{k_{-2}}{\overset{k_2[TF]}{\rightleftharpoons}} P_2 \tag{44}$$

the matrix U is:

$$U = \begin{bmatrix} -k_1 & k_{-1} & 0 \\ k_1 & -k_2 - k_{-1} & k_{-2} \\ 0 & k_2 & -k_{-2} \end{bmatrix}, \tag{45}$$

where we have set $[TF] = 1$ at the mid-boundary position. The matrix is of size 3×3 and can be diagonalized analytically in the general case. We define the following auxiliary variables:

$$\begin{cases} r = k_1k_2 + k_{-1}k_2 + k_{-1}k_{-2} \\ w = k_1 + k_2 + k_{-1} + k_{-2} \\ v = k_{-2} - k_1 - k_2 - k_{-1} \\ x = k_2 + k_{-2} - k_1 - k_{-1} \\ d = \sqrt{(k_1 + k_2 + k_{-1} + k_{-2})^2 - 4(k_1k_2 + k_1k_{-2} + k_{-1}k_{-2})} \end{cases} \tag{46}$$

and for the all-or-nothing case:

$$\alpha = \begin{pmatrix} 0 \\ 0 \\ 1 \end{pmatrix}, \tag{47}$$

the steady state probability is:

$$\langle f_P \rangle = \alpha^{\mathrm{T}} \overline{x} = \alpha^{\mathrm{T}} \begin{pmatrix} k_{-1}k_{-2}/r \\ k_{-2}k1/r \\ k_1k_2/r \end{pmatrix} = k_1k_2/r, \tag{48}$$

and the mean squared of the readout error is:

$$\begin{aligned}\langle f_P^2(T)\rangle = &\frac{2k_1k_2}{T^2r}\times \\ &\Bigg[\frac{T^2}{2r}\big(k_2k_{-2}+\frac{v^2-d^2}{4}\big) \\ &+\big(\frac{2T}{w-d}-\frac{4}{(w-d)^2}\big(1+e^{-(w-d)T/2}\big)\big)\frac{k_{-2}(x-d)}{2r-2w(w-d)+3(w-d)^2/2}\big) \\ &+\big(\frac{2T}{w+d}-\frac{4}{(w+d)^2}\big(1+e^{-(w+d)T/2}\big)\frac{k_{-2}(x+d)}{2r-2w(w+d)+3(w+d)^2/2}\Bigg]\end{aligned} \tag{49}$$

The analytically calculated readout relative error $CV_P(T) = \sqrt{\langle f_P^2(T)\rangle/\langle f_P\rangle^2 - 1}$ agrees with the numerical calculation for the $N = 2$ equilibrium model (Figure E).

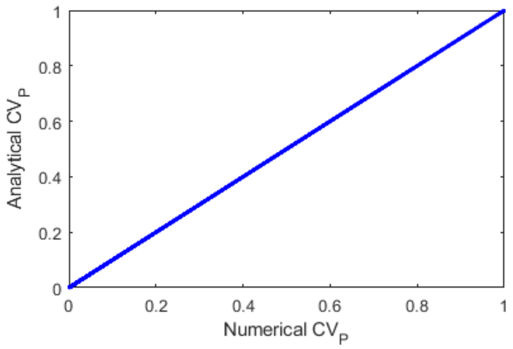

Figure E: **The analytical and numerical calculation of the relative error $CV_P(T)$ for the equilibrium model with $N = 2$.** Small discrepancies result from numerically finding the half-maximum expression point, which due to numerical precision is not exactly at $\langle f_P\rangle = 0.5$.

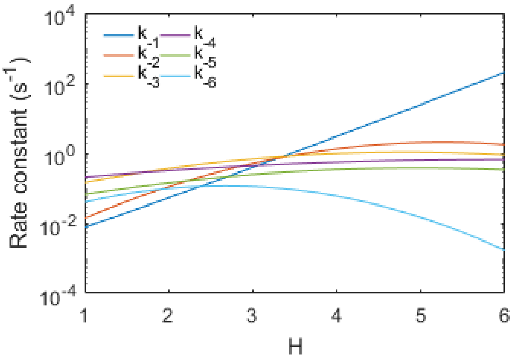

Figure F: **The unbinding rate constants k_{-i} yielding the highest readout error CV_P in nuclear cycle 12 of the equilibrium model with $N = 6$.** The optimal binding rate constants k_i are at their highest possible values $(N - i + 1)/\tau_{\text{bind}}$.

F

Positional resolution

Calculation of positional resolution

For each set of parameters $\overline{k}$, integration window T and nuclei distance ΔW, we generate 500 realizations of promoter activity at location $-\Delta W/2$ and $+\Delta W/2$. From each realization, we extract an individual gene readout $f_{-i} = f_P(-\Delta W/2)$ and $f_{+i} = f_P(+\Delta W/2)$, with $i = 1...500$. The distribution of the readout values at the two positions, F_+ and F_-, can be approximated marginally by the sample distribution of f_{+i} and f_{+j} (Figure G Panel A).

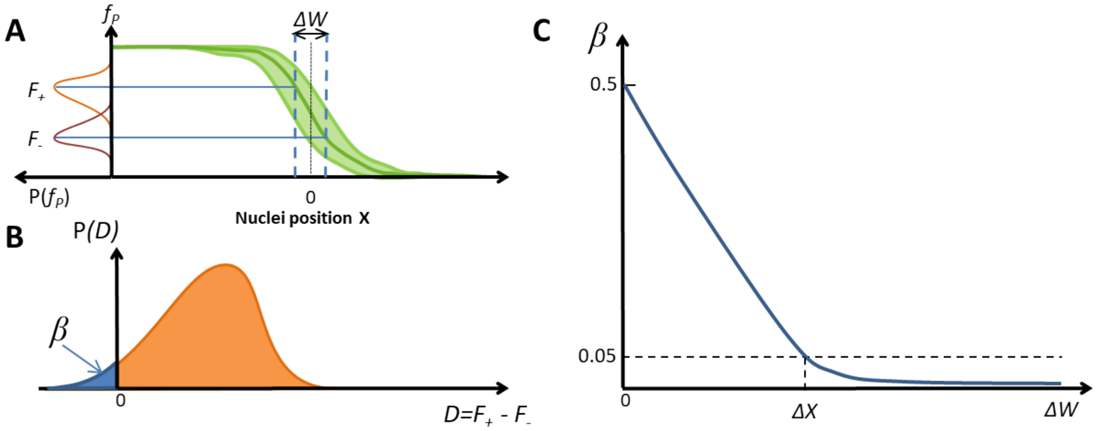

Figure G: **Finding the positional resolution**.
Panel A: The distribution of readout F_+ and F_- from two nuclei positioned at a distance of ΔW on opposite sides of the expression boundary.
Panel B: The coefficient $\beta = P(D \leq 0)$ is the risk of a nucleus wrongly predicting its position.
Panel C: ΔX is set as the smallest ΔW yielding a tolerable risk ($\beta \leq 5\%$).

The difference in the activity of two nuclei on opposite sides of the mid-boundary position is:

$$D = F_+ - F_-. \tag{50}$$

When D takes a non-negative value, we have a false negative result suggesting the anterior nucleus is not the anterior region. The probability β of getting such false negative samples is:

$$\beta = P(D \leq 0) = P(F_+ \leq F_-). \tag{51}$$

The value of β for each ΔW can be calculated numerically via the approximated distribution of F_+ and F_-. One observes that β decreases with increasing nuclei distance ΔW (Figure G Panel B). We set the risk tolerance level $\beta \leq 5\%$ to conclude whether the nuclei distance (ΔW) is large enough for any two nuclei to have different readout values. We define the positional resolution as such a value of ΔW that (Figure G Panel B):

$$\Delta X = \min\left(\Delta W | \beta \leq 0.05\right). \tag{52}$$

In practice, to determine the value of ΔX for each parameter set $\overline{k}$, we increase ΔW from $0 \times \lambda$ to $4 \times \lambda$ with an increment of $0.01 \times \lambda$ (λ is the TF gradient's decaying length, which is $\sim 100\mu_m$ or $\sim$ 20%EL), which corresponds to 0% to 80% of the embryo length. For each value of ΔW, the distribution of D and the value of β are computed from stochastic simulations of F_+ and F_- [10, 11]. As β also monotonically decreases with ΔW, ΔX is set as the first value of ΔW that gives $\beta \leq 0.05$ (Figure G Panel C).

When the nuclei readout is the average of M identical and independent identical single gene readouts ($F_+(j)$ and $F_-(j)$, for $j = 1..M$), the difference in the averaged readout at the two locations $-\Delta W/2$ and $+\Delta W/2$ is:

$$D_M = \frac{1}{M}\sum_{j=1}^{M}(F_+(j) - F_-(j)), \tag{53}$$

and β is calculated as $\beta = P(D_M \leq 0)$.

As M increases, it is expected that the difference in the averaged readout D_M at specific nuclei distance ΔW has reduced variance while maintaining the same mean level. This leads to a smaller risk level β and consequently smaller values of ΔX when compared with $M = 1$ case (Figure H).

Correlation between readout error and positional resolution

The correlation between the readout error and positional resolution given the same degree of pattern steepness is demonstrated in the $N = 6$ equilibrium model. We first find the randomized kinetic parameter sets that yield the Hill coefficient $H = 4$. The transcription readout error CV_P given $T = 400s$ varies between 0.12 and 1. Among these sets, we select 20 parameter sets yielding CV_P linearly spaced between 0.12 and 1 and calculate the positional resolution for each of the parameter set. The positional resolution (ΔX) as a function of readout error CV_P is shown in Figure I.

Positional resolution for a binomial readout

When the nuclear cycle is very short or when the promoter dynamics is very slow,the positional readout value any given position X depends only on the promoter activity state at the beginning of the nuclear cycle. At steady state, this activity state follows a Bernoulli distribution ($CV_P = 1$ as in Fig. 2 of the main manuscript) with a mean value $\langle f_P(X)\rangle$. We assume that the readout pattern can be well fitted with a sigmoid curve with a Hill coefficient H:

$$\langle f_P(X)\rangle = \frac{e^{HX}}{1 + e^{HX}}. \tag{54}$$

For the case $M = 1$ (single gene readout), at the anterior position $\Delta W/2$, the readout F_+ has a chance $f_P(\Delta W/2)$ to be 1. Similarly, at the opposite position $-\Delta W/2$, the readout F_- has a chance $1 - f_P(\Delta W/2)$ to be 1. Thus, the probability that two opposite nuclei falsely determine their position from their readout value is:

$$\begin{aligned}\beta &= P(F_+ \leq F_-) = 1 - P(F_+ > F_-) \\ &= 1 - P(F_+ = 1, F_- = 0) \\ &= 1 - f_P(\Delta W/2)^2.\end{aligned} \tag{55}$$

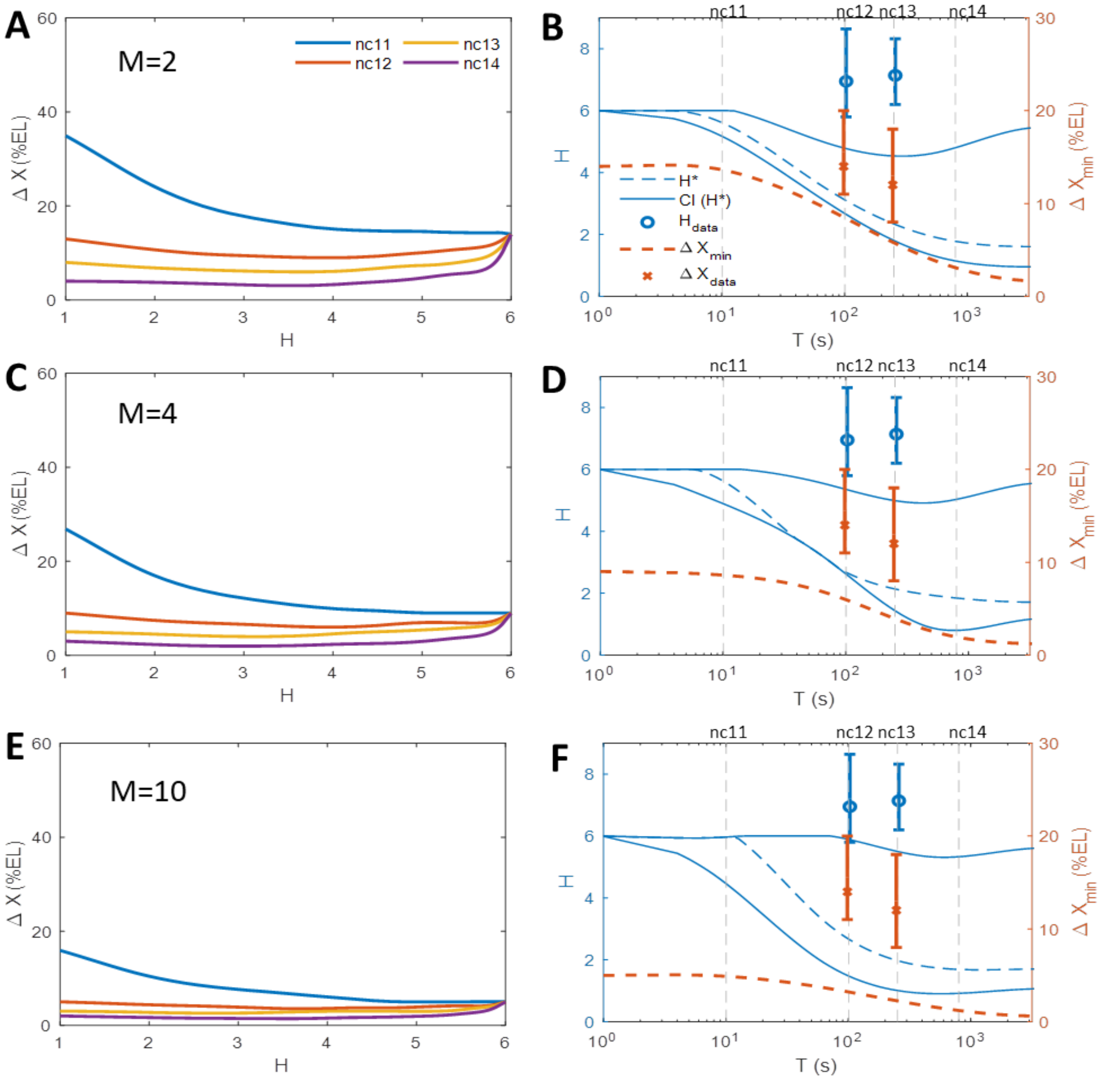

Figure H: **Positional resolution with varying single gene copy number per nuclei M in the equilibrium model.** Results for the "all-or-nothing" model with $N = 6$ binding sites and M equal 2 (A-B), 4 (C-D) and 10 (E-F).
(A,C,E) Positional resolution calculated from the equilibrium binding site model for varying boundary steepness H.
(B,D,F) The optimal Hill coefficients H^* that gives the minimal positional resolution (dashed blue line), the confidence interval CI(H^*) with 2 %EL tolerance (solid blue lines) and the lowest value of the positional resolution ΔX_{min} (orange dashed line), for varying T. The theoretical results are compared to the empirical Hill coefficient H_{data} (blue crosses) and positional resolution ΔX_{data} (orange crosses) extracted from MS2-MCP live imaging data.

When we increase ΔW from 0 until β reaches 5%, we find the positional resolution $\Delta X = \Delta W$. Therefore:

$$1 - f_P(\Delta X/2)^2 = 0.05, \tag{56}$$

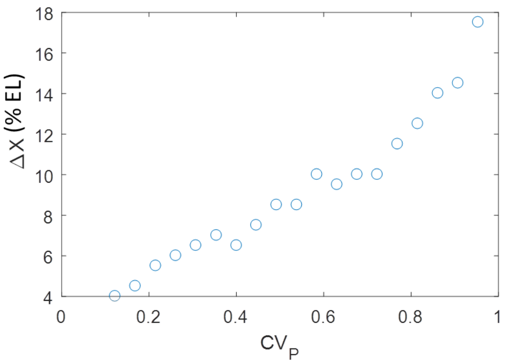

Figure I: **The correlation between the readout error (CV_P) and positional resolution (ΔX).** Demonstrated with $N = 6$ and $H = 4$ and $T = 400s$. The kinetic parameters are selected so as to generate the same Hill coefficient $H = 4$ and readout error CV_P linearly spaced between its bound 0.12 and 1.

or

$$\begin{aligned} f_P(\Delta X/2) &= \sqrt{1-0.05} \\ &= \frac{e^{H\Delta X/2}}{1+e^{H\Delta X/2}} \\ &= 1 - \frac{1}{1+e^{H\Delta X/2}}, \end{aligned} \tag{57}$$

which gives

$$\Delta X = \frac{2}{H} ln(\frac{1}{1-\sqrt{0.95}} - 1) \approx \frac{7.3}{H}. \tag{58}$$

and the value of ΔX in $\%EL$ unit is:

$$\Delta X \approx \frac{7.3}{H} * 20\%EL = \frac{146\%EL}{H} \tag{59}$$

In the case $M > 1$, the positional readout follows a scaled binomial distribution:

$$\begin{aligned} F_+ &\sim \frac{1}{M} B(M, f_P(\Delta W/2)), \\ F_- &\sim \frac{1}{M} B(M, f_P(-\Delta W/2)), \end{aligned} \tag{60}$$

and the value of ΔX is calculated numerically by solving $\beta = P(F_+ \leq F_-) = 0.05$.

Positional resolution

To calculate the positional resolution of the *hb* pattern in live fly embryos, for each position along the embryo AP axis, we collect the readout of all nuclei in this position (with a bin width of 5% of the embryo length). We then find the distribution of the difference $P(F_+ - F_-)$ at position $+\Delta W/2$ and $-\Delta W/2$ from the pattern's boundary, with ΔW increasing from 0 %EL. Assuming that this difference follows a normal distribution, we calculate the risk β and its confidence interval (p-value=0.05) (Figure J). By inspecting when the risk value is tolerable ($\leq$ 5 %), we find $\Delta X \sim$ 14%EL (confidence interval from 11% EL to 20% EL) in nuclear cycle 12 and $\Delta X \sim$ 12% EL (confidence interval from 8% EL to 18% EL) in nuclear cycle 13.

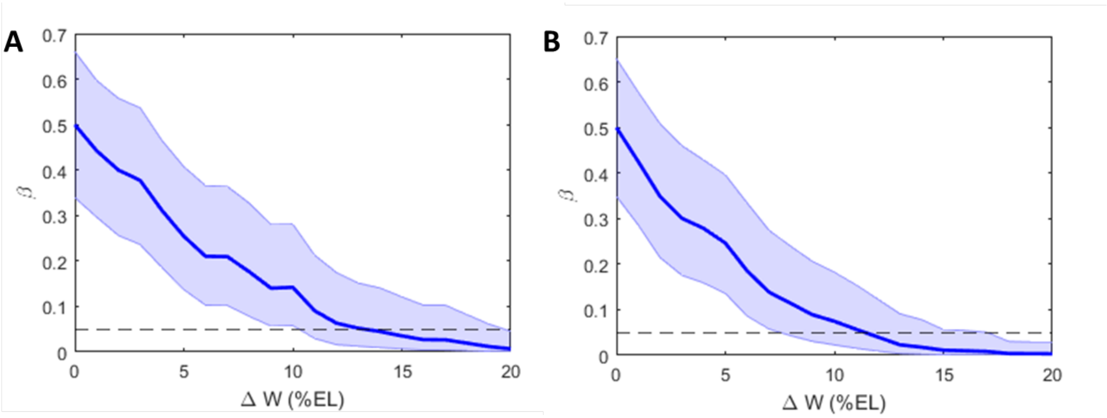

Figure J: **The risk factor value β as a function of ΔW for the *hb* proximal promoter (solid line)**, plotted with the confidence interval (shaded) with p-value=0.05. The dashed black line indicates the tolerable risk $\beta = 0.05$. Panel A: Nuclear cycle 12 (8 embryos). Panel B: Nuclear cycle 13 (4 embryos).

G

Analysis of the non-equilibrium model

The steady state of the non-equilibrium models can be a limit-cycle instead of simple fix points. Therefore, to assess whether the system has reached steady state we consider both the probability of the promoter to be active $\mu_P(T, 0)$ like we did in steady state and the derivative of this probability over time:

$$\mu_{SS} = |\mu_P(T, 0) - 0.5| + |\frac{\partial \mu_P(T, 0)}{\partial T}|. \tag{61}$$

If the system has reached steady state at time T, at the mid-embryo position, we expect $\mu_P(T, 0)$ to be equal 0.5 and its derivative term to be 0, and thus $\mu_{SS} \approx 0$. If $\mu_{SS} > 0$ the system has not yet reached steady state.

Full non-equilibrium model with $N = 3$ binding sites

We first investigate the "all-or-nothing" non-equilibrium model with 3 OS ($N = 3$). Figure K shows that the model is able to achieve a higher steepness ($H \leq 2N - 1 = 5$) than that with the equilibrium model ($H \leq N = 3$), as described in [2]. Similarly to the equilibrium model we observe a tradeoff between the pattern steepness H, readout error and pattern formation time. In the case of the steepest pattern ($H = 5$), the pattern is not yet formed ($\mu_{SS} = 0.5$) and the noise is at its highest value ($CV_P = 1$).

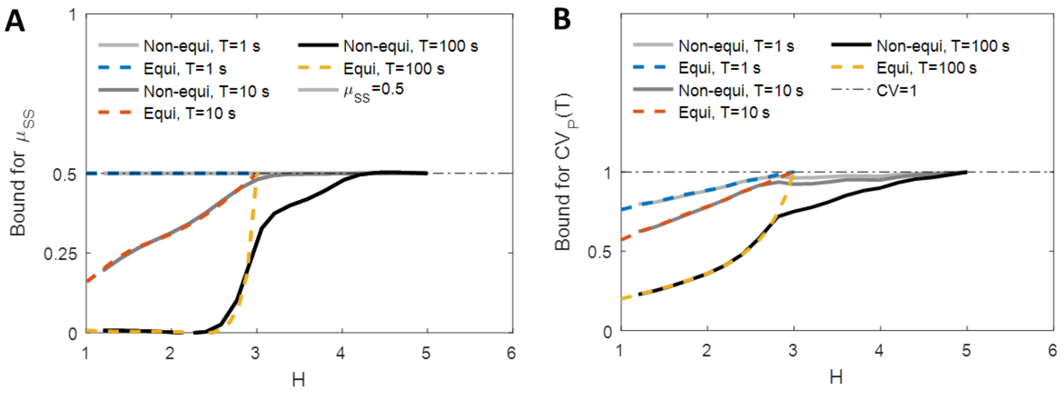

Figure K: **Readout error of a pure non-equilibrium model for $N = 3$.**
Panel A: The lower bounds for μ_{SS} from the non-equilibrium model (grey solid line), for varying values of T, computed from 3×10^5 data points. Also shown are the bounds for equilibrium model (colored dashed lines).
Panel B: The lower bounds for readout error $CV_P(T)$ for the non-equilibrium model (grey solid lines) for varying value of T computed from 3×10^5 data points. Also shown are the bounds for the equilibrium model (colored dashed lines).

Hybrid non-equilibrium model with $N = 3$ binding sites

We expand the non-equilibrium model to $N = 6$. However, we do not use a full model (as in Figure A) due to the very large numbers of micro-states ($2^6 = 64$) and possible transitions ($6 \cdot 2^6 =$

396), which makes numerical optimization of the parameters numerically costly. Instead, we opt to use a hybrid model with 2 OS arrays. The first array contains 3 identical OS, the interactions of which with the TF are at equilibrium (as in Eq. 4). The second array contains 3 OS, the interactions of which with the TF are out of equilibrium (as in Figure A). To include cooperativity between the binding sites and decrease the computational time of the numerical parameter optimization we further assume the dynamics of the two arrays are not independent: TF can only interact with the first OS array when the second OS array is completely free, and TF can only interact with the second OS array when the first array is fully bound.

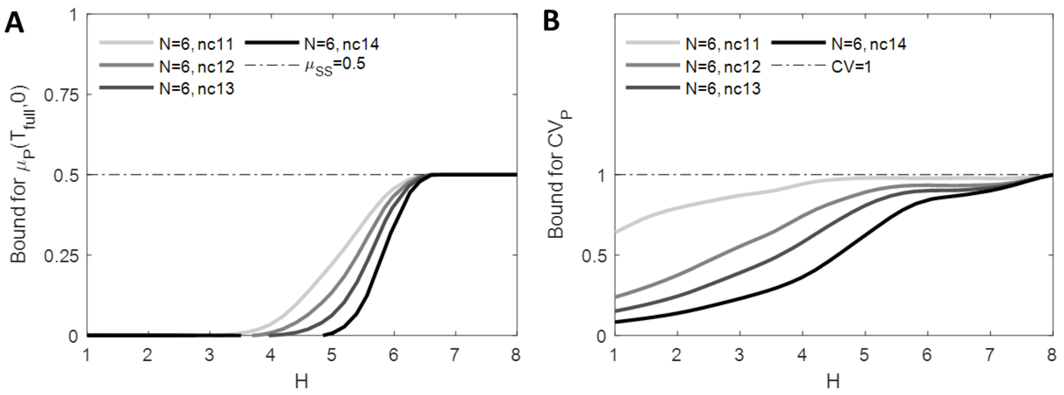

Figure L: **Readout error of the hybrid non-equilibrium model with $N = 6$, three equilibrium and three non-equilibrium OS**.
Panel A: The lower bounds for μ_{SS} for a hybrid non-equilibrium model (grey solid lines) for varying values of T computed from 10^6 data points.
Panel B: The lower bounds for the readout error $CV_P(T)$ for a hybrid non-equilibrium model (grey solid lines), for varying values of T computed from 3×10^5 data points.

The hybrid model is able to achieve a steepness of 8 (Figure L), as expected from equilibrated activity of 3 OS and non-equilibrated activity of 3 OS. The tradeoff between the pattern steepness H and the readout error and pattern formation time still holds. Note that the hybrid model is not nested in the equilibrium model.

The positional resolution for the hybrid model with varying nuclear cycle is shown in Figure M and the optimal steepness with varying interphase duration T is plotted in Fig. 4C of the main text.

Analysis of the "K-or-more" case

The results concerning the "K-or-more" case is shown in Figure N, from which qualitatively similar observations as in the "all-or-nothing" case can be drawn.

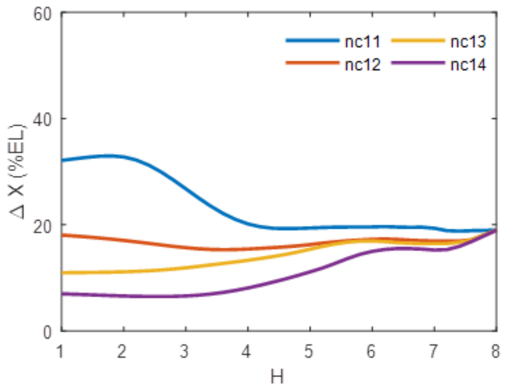

Figure M: **Positional resolution in the $N = 6$ hybrid non-equilibrium model.** The results are shown for the "all-or-nothing" case with $N = 6$, $M = 1$. Positional resolution calculated for a hybrid non-equilibrium binding site model for varying boundary steepness H.

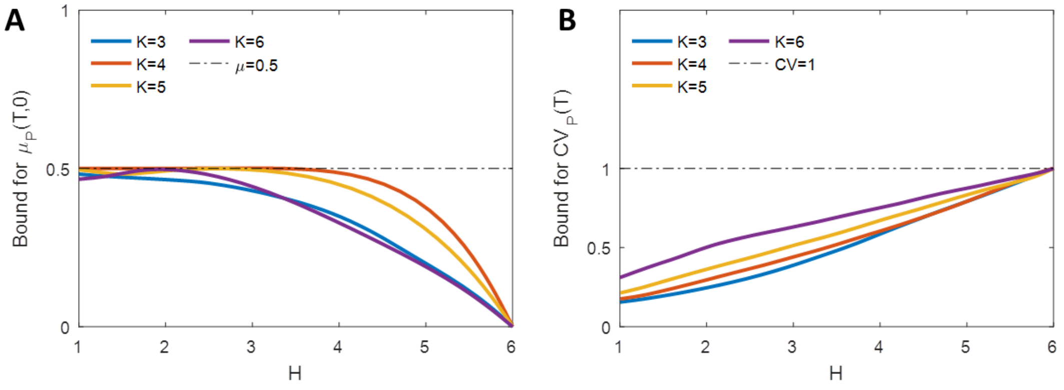

Figure N: **The "K-or-more" case is qualitatively similar to the "all or nothing" case.** The results are shown with $N = 6$ binding sites and an interphase duration of $T = 500$ s. $K = 6$ corresponds to the "all or nothing" case. Each curve is computed from ~ 20000 data points.

Panel A: The lower bound for the mean promoter activity level at the boundary position $\mu_P(T, 0)$, for different K values (solid colored lines), as a function of pattern sharpness H. The $\mu = 0.5$ line (dashed line) indicates the steady-state value.

Panel B: The lower bound for readout error $CV_P(T)$ for different K values (solid colored lines) as a function of pattern sharpness H. Also shown is the upper bound for the noise, $CV = 1$.

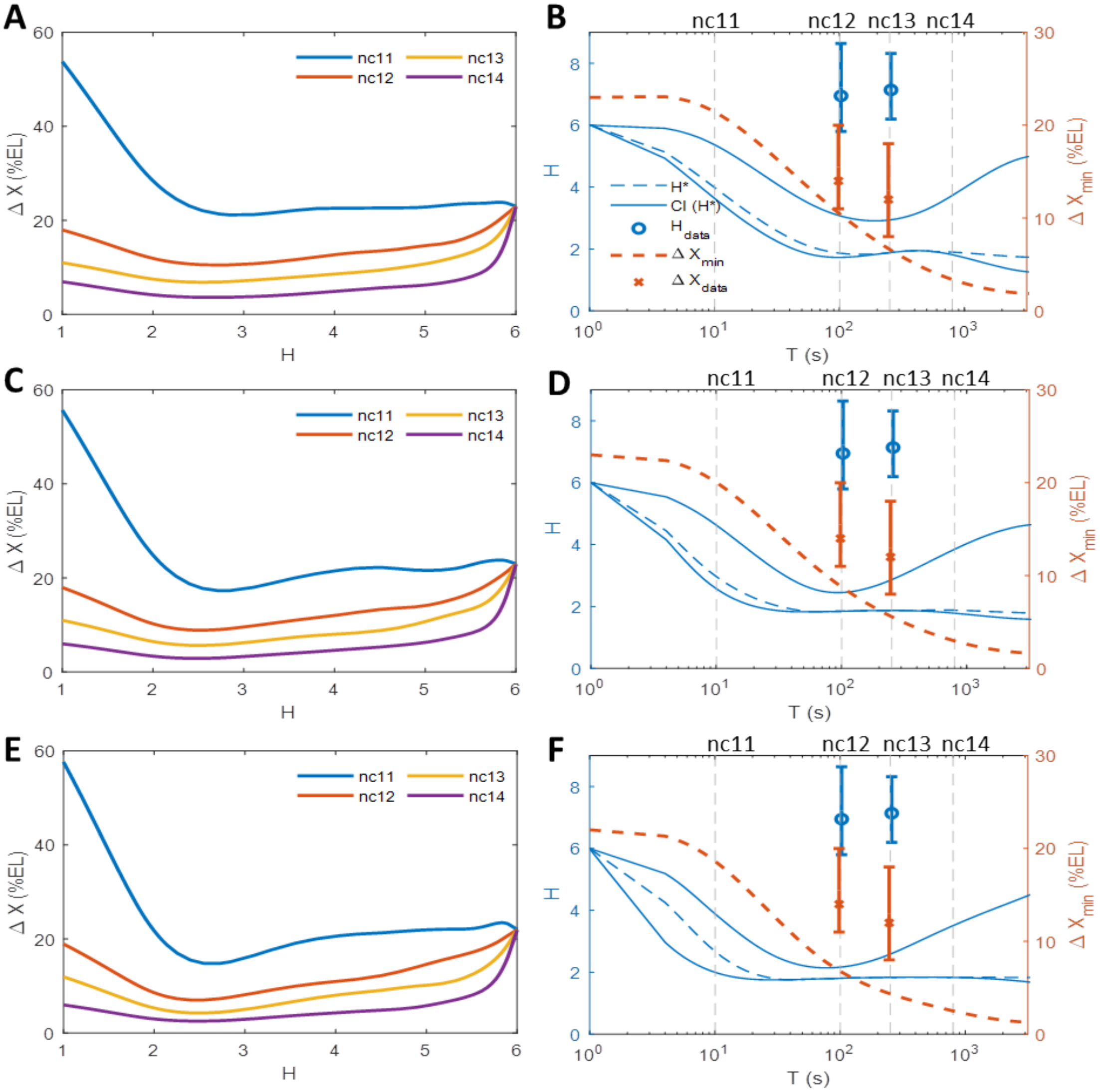

Figure O: **Positional resolution in the "K-or-more" case, with varying K.** The results are shown with $N = 6$ binding sites and K equal 5 (A-B), 4 (C-D), 3 (E-F). $M = 1$.
(A,C,E) Positional resolution calculated from the equilibrium binding site model for varying boundary steepness H.
(B,D,F) The optimal Hill coefficients H^* that gives the lowest value of the positional resolution (dashed blue line), the confidence interval CI(H^*) with 2 %EL tolerance (solid blue lines) and the lowest value of the positional resolution ΔX_{min} (orange dashed line), for varying T. The theoretical results are compared to the empirical Hill coefficient H_{data} (blue crosses) and positional resolution ΔX_{data} (orange crosses) extracted from MS2-MCP live imaging data.

H

Transcription pattern formed by two transcription factor gradients

We investigate the transcription pattern formation under the independent regulation of two transcription factor gradients: an anterior activator TF (modeled as above) and a repressor TF', which is concentrated at either the posterior (e.g. Cad protein) or mid-embryo (Cic protein).

The transcription factors regulate the target gene via interactions with the activator binding site array A and repressor binding site array B, each with N and L identical binding sites respectively:

$$A_0 \underset{k_{-1}}{\overset{k_1[TF]}{\rightleftharpoons}} A_1 \underset{k_{-2}}{\overset{k_2[TF]}{\rightleftharpoons}} A_2 ... \underset{k_{-N}}{\overset{k_N[TF]}{\rightleftharpoons}} A_N, \tag{62}$$

$$B_0 \underset{k'_{-1}}{\overset{k'_1[TF']}{\rightleftharpoons}} B_1 \underset{k'_{-2}}{\overset{k'_2[TF']}{\rightleftharpoons}} B_2 ... \underset{k'_{-L}}{\overset{k'_L[TF']}{\rightleftharpoons}} B_L. \tag{63}$$

We call α and γ the vectors indicating which states are ON (the ith elements of α and γ respectively indicate whether A_i or B_i is an active or an inactive state). We consider the "all-or-nothing" model for the activator ($\alpha = [00...1]^T$) and a "zero-or-nothing" model for repressor ($\gamma = [10...0]^T$).

In each nuclear cycle of duration T, A and B produce time traces $a(t)$ and $b(t)$. The mean activity levels $A(T)$ and $B(T)$ are:

$$A(T) = \frac{1}{T}\int_{t=0}^{T} \alpha^\intercal a(t)dt \tag{64}$$
$$B(T) = \frac{1}{T}\int_{t=0}^{T} \gamma^\intercal b(t)dt.$$

We consider the promoter to be active when both the binding arrays are active:

$$P_{\text{active}} = \alpha^\intercal a(t)\gamma^\intercal b(t). \tag{65}$$

The promoter readout is given by:

$$f_P(T) = \frac{1}{T}\int_{t=0}^{T} \alpha^\intercal a(t)\gamma^\intercal b(t)dt. \tag{66}$$

At a given position, the two arrays have rate matrices U_a and U_b respectively. We call $\overline{x}_a$ and $\overline{x}_b$ the steady state solution of $U_a x = 0$ and $U_b x = 0$ respectively.

Scenario 1: posterior repressor

In the first scenario, the repressor has an exponentially decay gradient from the posterior, mirroring the anterior gradient:

$$[TF'] = e^{-X}. \tag{67}$$

We select $N = 6$, $L = 6$.

The pattern steepness

The mean promoter readout is given by:

$$\langle f_P(T)\rangle = \frac{1}{T}\int_{t=0}^{T} \langle \alpha^\intercal a(t)\gamma^\intercal b(t)\rangle dt. \tag{68}$$

Given that $a(t)$ and $b(t)$ are independent and assuming the system is at steady-state, we have:

$$\begin{aligned}\langle f_P(T)\rangle &= \frac{1}{T}\int_{t=0}^{T} \langle \alpha^\intercal a(t)\rangle\langle\gamma^\intercal b(t)\rangle dt \\ &= \langle A(T)\rangle\langle B(T)\rangle,\end{aligned} \tag{69}$$

and at the promoter activity boundary ($X = 0, \langle P(T)\rangle = 0.5$) the steepness of the promoter activity pattern is:

$$\begin{aligned}H_P &= 4(\frac{\partial\langle f_P(T)\rangle}{\partial X})_{X_0} \\ &= 4(\frac{\partial(\langle A(T)\rangle\langle B(T)\rangle)}{\partial X})_{X_0} \\ &= 4\langle B(T)\rangle(\frac{\partial\langle A(T)\rangle}{\partial X})_{X_0} + 4\langle A(T)\rangle(\frac{\partial\langle B(T)\rangle}{\partial X})_{X_0} \\ &= \langle B(T)\rangle H_A + \langle A(T)\rangle H_B.\end{aligned} \tag{70}$$

Given Eq. 70, we have:

$$\begin{aligned}H_P &\leq 4N\langle B(T)\rangle\langle A(T)\rangle(1-\langle A(T)\rangle) + 4L\langle A(T)\rangle\langle B(T)\rangle(1-\langle B(T)\rangle) \\ &= 4\langle P(T)\rangle(N + L - N\langle A(T)\rangle - L\langle B(T)\rangle) \\ &= 2(N + L - N\langle A(T)\rangle - L\langle B(T)\rangle)\end{aligned} \tag{71}$$

In the case $N = L$, we have the upper bound for H_P:

$$\begin{aligned}H_P &\leq 4N - 2N(\langle A(T)\rangle + \langle B(T)\rangle) \\ &\leq 4N - 4N\sqrt{\langle A(T)\rangle\langle B(T)\rangle} \\ &= 4N(1 - \frac{1}{\sqrt{2}}) \approx 1.17N.\end{aligned} \tag{72}$$

The equality in Eq. 72 occurs when $\langle A(T)\rangle = \langle B(T)\rangle = \frac{1}{\sqrt{2}}$. From Eq. 72, we found that having two independent binding site arrays does not yield significant higher pattern steepness than that achievable with a single array.

Mean promoter activity out-of-steady-state

We call a_0 and b_0 the initial state of the OS arrays A and B. At the end of the interphase of duration T, the mean probability that the promoter is active is:

$$\begin{aligned}\langle f_P(T)\rangle &= \langle \alpha^\intercal a(T)\rangle\langle\gamma^\intercal b(T)\rangle \\ &= (\alpha^\intercal e^{U_a T} a_0)(\gamma^\intercal e^{U_b T} b_0).\end{aligned} \tag{73}$$

The upper bound for the mean promoter activity level at the end of each nuclear cycle interphase is shown in Figure P Panel A for $N = L = 6$.

The readout error

Assuming the system is at steady state, the mean square of the readout:

$$\begin{aligned}\langle (f_P(T))^2 \rangle &= 1/T^2 \int_{u=0}^{T} du \int_{s=0}^{T} ds \langle p(u)p(s) \rangle \\ &= 1/T^2 \int_{u=0}^{T} du \int_{s=0}^{T} ds \langle \alpha^\intercal a(u) \alpha^\intercal a(s) \rangle \langle \gamma^\intercal b(u) \gamma^\intercal b(s) \rangle \\ &= 2/T^2 \int_{s=0}^{T} ds (T-s) \alpha^\intercal e^{U_a |u-s|} \left(\overline{x}_a . \alpha \right) \gamma^\intercal e^{U_b |u-s|} \left(\overline{x}_b . \gamma \right).\end{aligned} \tag{74}$$

is calculated numerically given the transition matrices U_a and U_b and used to calculate the promoter activity readout relative error (the . here corresponds to term by term multiplication of the vectors coordinates).

The lower bound for promoter activity readout error after each nuclear cycle is shown in Figure P Panel B for $N = L = 6$.

When H approaches its maximal value and τ_{active} goes to infinity, the integrated activity of each transcription factor becomes binomial. If p is the probability of the activator binding array being full bound and and q the probability of the repressor binding array being free, we have $\langle f_P(T) \rangle = pq$ and $CV_P(T) = (1 - pq)/pq$. If $pq = 1/2$ we recover $CV_P(T) = 1$. Consistently, the limit of Eq. 74 when all non-zero eigenvalues of U_a and U_b go to $-\infty$ yields the same result.

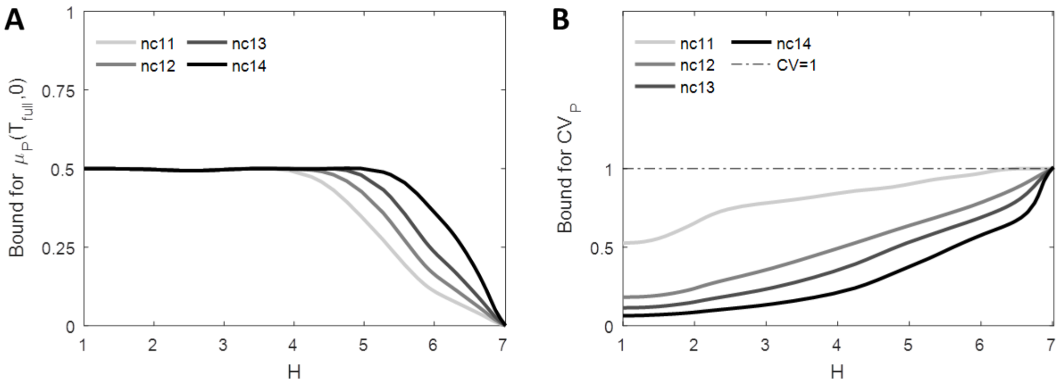

Figure P: **The trade-off between pattern steepness (H), pattern formation time and readout error in the case of transcription regulation by two transcription factors**. Results for $N = L = 6$. $M = 1$. Each curve is computed from > 20000 data points.
Panel A: The lower bounds for the mean promoter activity level at the boundary position μ_{SS} for varying nuclear cycles.
Panel B: The lower bounds for readout error $CV_P(T)$ for varying nuclear cycles. Also plotted is the dashed line $CV = 1$.

Scenario 2: mid-embryo repressor

In the second scenario, the repressor is concentrated at the boundary position. The repressor gradient is modelled as a Gaussian curve with standard deviation $\sigma = 1.25$ (equivalent to 25 %EL).

$$[TF'] = e^{\frac{X^2}{\sigma^2}}. \tag{75}$$

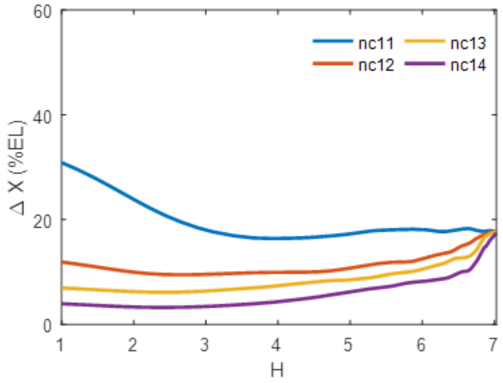

Figure Q: **Positional resolution in the case of transcription regulation by two mirror transcription factor gradients**. The results are shown for $N = 6$, $L = 6$.
Panel A: Positional resolution calculated from the equilibrium binding site model with randomized kinetic parameters that give different values of the expression profile steepness H for $M = 1$. The colored lines show the results for parameters that give the smallest readout error CV_P from a set of randomized parameters for the steady-state window T in nc 11-14.
Panel B: The range of optimal Hill coefficients H^* (dashed blue line) that yield the lowest value of the positional resolution (obtained from panel A) as a function of the steady-state readout duration T. The error bars correspond to 95% confidence intervals.

TF can interact with the promoter via $L = 1$ binding site, corresponding to the number of known Cic binding sites found on *hb* promoter [12]. For simplicity, we consider $k'_1 = k'_{-1} = 1$.

At the boundary position, given the local flat repressor concentration, the pattern steepness is dependent on the regulation function of only the activator:

$$\frac{\partial \langle f_P(T) \rangle}{\partial X} = \frac{\partial \langle A(T) \rangle \langle B(T) \rangle}{\partial X} = \frac{\partial \langle A(T) \rangle}{\partial X} = H_A \leq N \tag{76}$$

We plot the positional resolution of the readout in Figure R. The kinetic parameters k_i and k_{-i} are selected so as to minimize the readout error from 6 activator binding sites for varying pattern steepness.

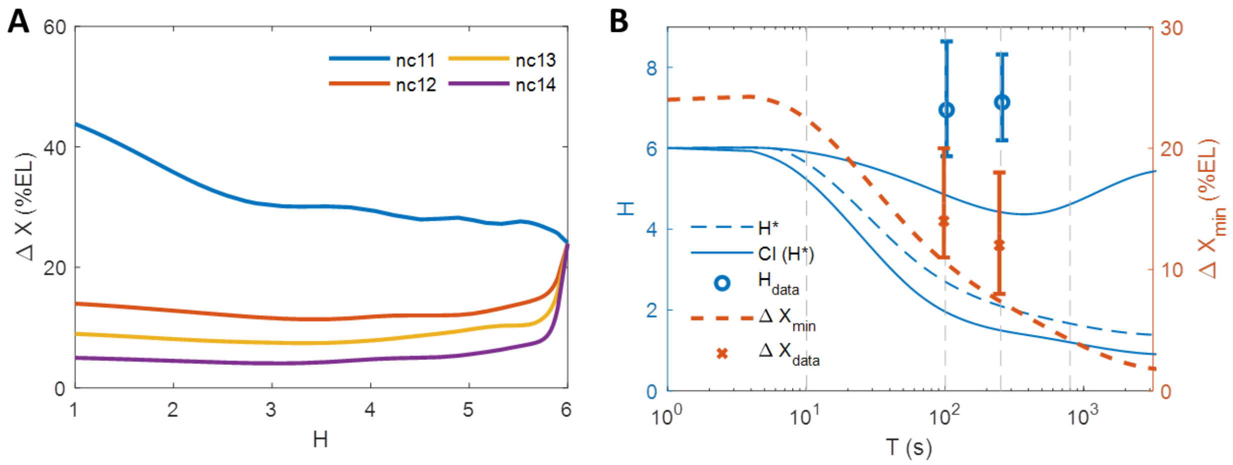

Figure R: **Positional resolution in the case of transcription regulation by an anterior activator and a mid-embryo repressor**. The results are shown for $N = 6$, $L = 1$.
Panel A: Positional resolution calculated from the equilibrium binding site model with randomized kinetic parameters that give different values of the expression profile steepness H for $M = 1$. The colored lines show the results for parameters that give the smallest readout error CV_P from a set of randomized parameters for the steady-state window T in nc 11-14.
Panel B: The range of optimal Hill coefficients H^* (dashed blue line) that yield the lowest value of the positional resolution (obtained from panel A) as a function of the steady-state readout duration T. The error bars correspond to 95% confidence intervals.

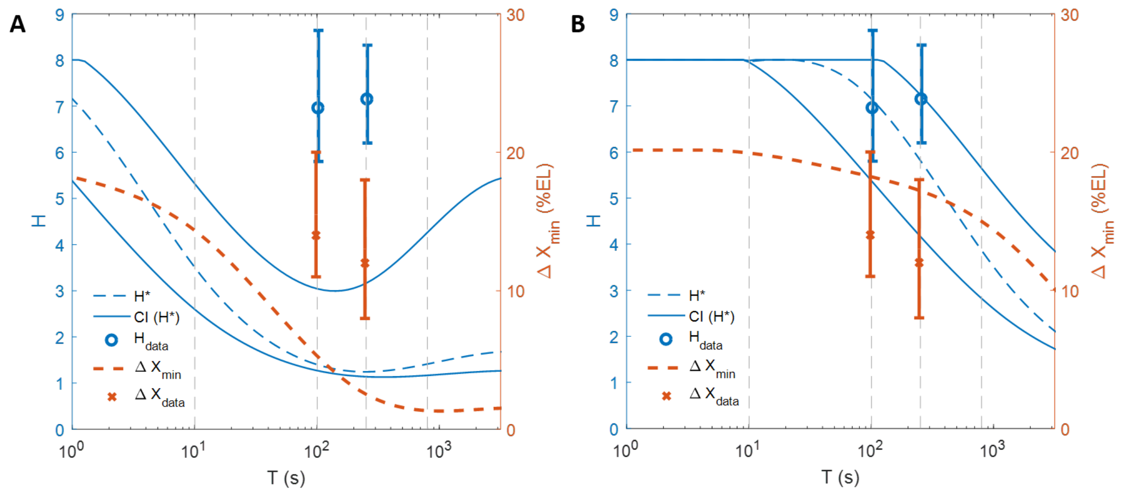

Figure S: **Optimal positional resolution with different values of τ_{bind}.** Panel A $\tau_{\text{bind}} = 0.4s$. Panel B: $\tau_{\text{bind}} = 40s$. The optimal positional resolution and Hill coefficient are calculated for the hybrid non-equilibrium model $N = 6$, $M = 1$ in the "all-or-nothing" case.

Expression pattern of proximal *hb* promoter in live *Drosophila* embryos

We observe the transcription dynamics of a 700bp *hb* P2 minimal promoter using the RNA-tagging MS2-MCP system [13, 14]. Here, the nascent RNAs in each transcription loci are visualized as bright spots under the confocal microscope, due to the co-localization of fluorescent tagged MS2-GFP molecules [15]. The data for the analysis can be obtained in Lucas *et al.*, 2013 [16].

The pattern steepness

From each nucleus, we obtain a single gene readout f_P – the total spot intensity observed during the interphase. We fit the readout values along the AP axis with a sigmoid curve using least-mean-square and infer the Hill coefficient (Figure T). The inferred Hill coefficients in nuclear cycle 12 is from 6.9, with the confidence interval from 5.80 to 8.64 (p-value=0.05). In nuclear cycle 13, the Hill coefficient is 7.1,with the confidence interval from 6.20 to 8.32 (p-value=0.05).

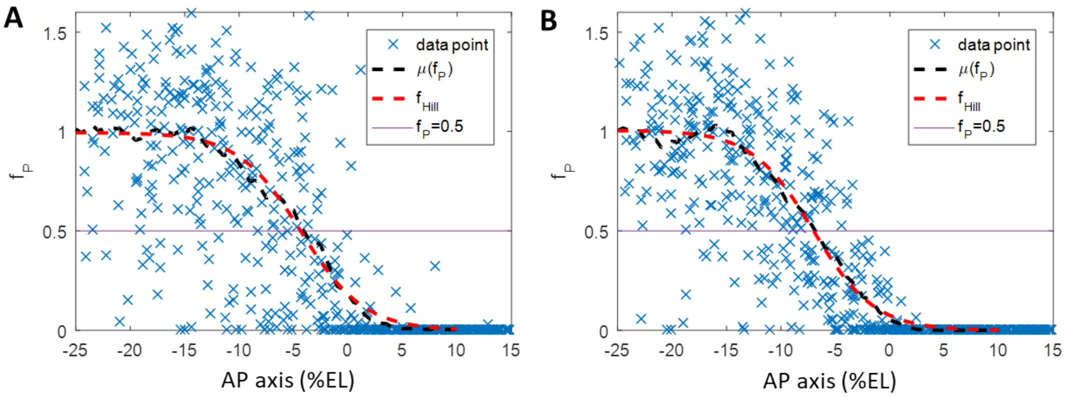

Figure T: **The transcription readout pattern by *hb* proximal promoter along AP axis.** normalized fluorescence (blue crosses), the mean readout $\langle f_P \rangle$ (dashed black line), the fitted Hill function (dashed red line) and $f_P = 0.5$ (solid yellow line) as a function of nuclei position. The normalized fluorescence and mean expression curves are normalized by the fitted Hill function's maximum value. Panel A: Nuclear cycle 12 (8 embryos). Panel B: Nuclear cycle 13 (4 embryos).

Transcription readout error

From the fitted sigmoid curve, we identify the *hb* pattern boundary position at $\sim -5\%$ EL from the middle of the embryo for both nc 12 and nc 13. The readout distributions around the boundary (within $\pm 2.5\%$ EL) are shown in Figure U. From the distributions, we calculate readout errors CV_P to be ~ 0.82 in nc 12 and ~ 0.69 in nc 13.

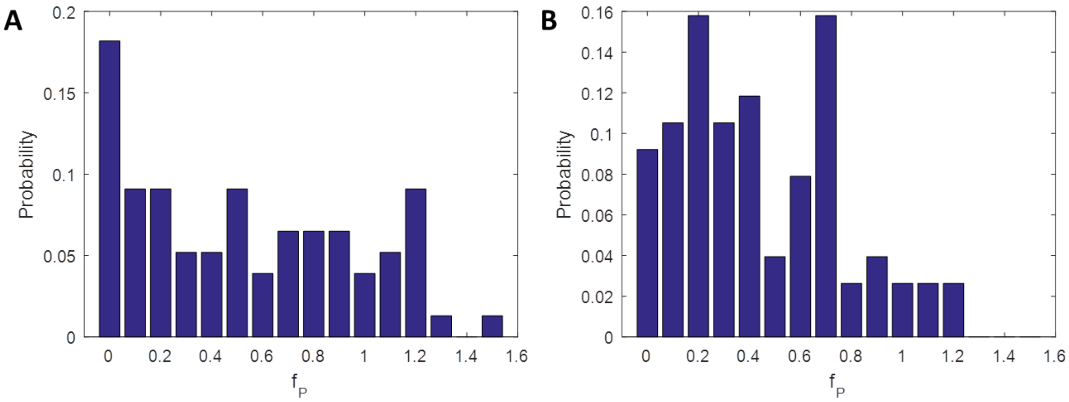

Figure U: **Distributions of *hb* transcription readout at mid-boundary position** in nuclear cycle 12 (Panel A, from 8 embryos) and nuclear cycle 13 (Panel B, from 4 embryos).

τ_{bind} to achieve experimentally observed pattern steepness and positional resolution

We vary the TF searching time for a single binding site τ_{bind} so as to fix the pattern steepness to the experimentally measured $H_{\mathrm{data}} = 7$ and find the minimal value of the positional resolution ΔX given this constraint. ΔX as a function of τ_{bind} in each cycle is shown in Figure V. From Figure V, we find that values close to the experimentally observed positional resolution ($\Delta X_{\mathrm{data}} \sim 14\%$ EL in nc 12 and $\Delta X_{\mathrm{data}} \sim 12\%$ EL in nc 13) and pattern steepness ($H_{\mathrm{data}} = 7$) can be achieved simultaneously with small τ_{bind} ($\tau_{\mathrm{bind}} \sim 1.2s$ in nc12 and $\tau_{\mathrm{bind}} \sim 0.12s$ in nc13).

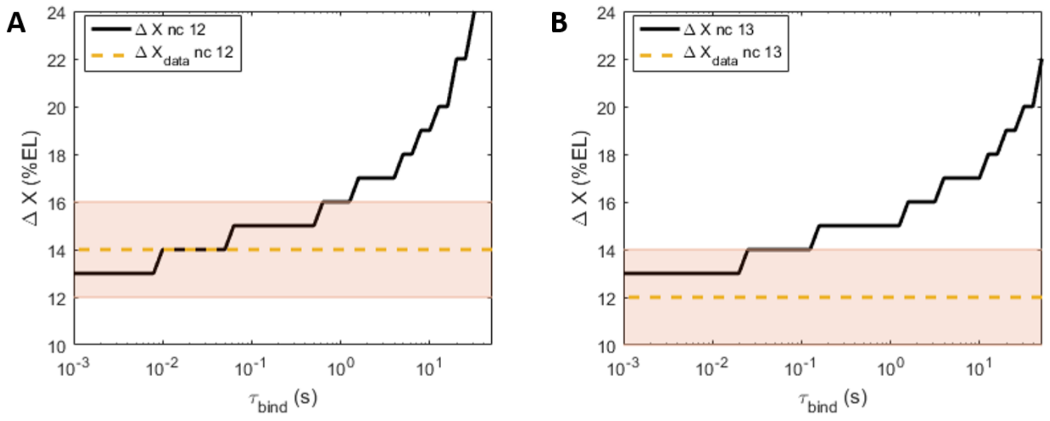

Figure V: **Positional resolution ΔX of the hybrid non-equilibrium model as a function of TF searching time for a binding site τ_{bind} for nc 12 (Panel A) and nc 13 (Panel B)**. The kinetic parameters are selected to achieve the experimentally observed Hill coefficient $H_{\mathrm{data}} = 7$. Also shown are the observed positional resolution ΔX_{data} in nc 12 (dashed green line in A) and nc 13 (dashed green line in B) and the 95% confidence intervals (shaded stripe in Panel A and B).

References

1. Gunawardena J. A linear framework for time-scale separation in nonlinear biochemical systems. PLoS ONE. 2012;7(5).

2. Estrada J, Wong F, DePace A, Gunawardena J. Information integration and energy expenditure in gene regulation. Cell. 2016;166(1):234–244.

3. Berg HC, Purcell EM. Physics of chemoreception. Biophysical Journal. 1977;20(2):193–219.

4. Abu-Arish A, Porcher A, Czerwonka A, Dostatni N, Fradin C. High mobility of bicoid captured by fluorescence correlation spectroscopy: Implication for the rapid establishment of its gradient. Biophysical Journal. 2010;99(4):33–35.

5. Porcher A, Abu-Arish A, Huart S, Roelens B, Fradin C, Dostatni N. The time to measure positional information: maternal hunchback is required for the synchrony of the Bicoid transcriptional response at the onset of zygotic transcription. Development. 2010;137(16):2795–804.

6. Gregor T, Tank DW, Wieschaus E, Bialek W. Probing the limits to positional information. Cell. 2007;130(1):153–164.

7. Xu H, Sepúlveda LA, Figard L, Sokac AM, Golding I. Combining protein and mRNA quantification to decipher transcriptional regulation. Nature methods. 2015;12(8):739–42.

8. Lopes FJ, Spirov AV, Bisch PM. The role of Bicoid cooperative binding in the patterning of sharp borders in Drosophila melanogaster. Developmental Biology. 2011;72(2):181–204.

9. Gregor T, Wieschaus E, McGregor AP, Bialek W, Tank DW. Stability and nuclear dynamics of the Bicoid morphogen gradient. Cell. 2007;130(1):141–152.

10. Gillespie DT. A general method for numerically simulating the stochastic time evolution of coupled chemical reactions. Journal of Computational Physics. 1976;22(4):403–434.

11. Lloyd-Price J, Gupta A, Ribeiro AS. SGNS2: A compartmentalized stochastic chemical kinetics simulator for dynamic cell populations. Bioinformatics. 2012;28(22):3004–3005.

12. Löhr U, Chung HR, Beller M, Jäckle H. Antagonistic action of Bicoid and the repressor Capicua determines the spatial limits of Drosophila head gene expression domains. Proceedings of the National Academy of Sciences. 2009;106(51):21695–21700.

13. Lucas T, Ferraro T, Roelens B, Chanes JDLH, Walczak AM, Coppey M, et al. Live imaging of Bicoid-dependent transcription in Drosophila embryos. Current Biology. 2013;23(21):2135–2139.

14. Garcia HG, Tikhonov M, Lin A, Gregor T. Quantitative imaging of transcription in living Drosophila embryos links polymerase activity to patterning. Current Biology. 2013;23(21):2140–2145.

15. Bertrand E, Chartrand P, Schaefer M, Shenoy SM, Singer RH, Long RM. Localization of ASH1 mRNA particles in living yeast. Molecular Cell. 1998;2(4):437–445.

16. Lucas T, Tran H, Perez Romero CA, Mathieu Coppey, Fradin C, Walczak AM, et al. Precision in a rush: trade-offs between positioning and steepness of the hunchback expression pattern. bioRxiv. 2018;305532.
